# Prevalence of Musculoskeletal and Metabolic Disorders in Kidney Transplant Recipients: A Systematic Review and Meta-Analysis

**DOI:** 10.3389/ti.2024.12312

**Published:** 2024-04-24

**Authors:** Álvaro Herreros-Carretero, Carlos Berlanga-Macías, Vicente Martínez-Vizcaíno, Ana Torres-Costoso, Carlos Pascual-Morena, Luis Enrique Hernández-Castillejo, Irene Sequí-Domínguez, Miriam Garrido-Miguel

**Affiliations:** ^1^ Facultad de Enfermería, Universidad de Castilla-La Mancha, Albacete, Spain; ^2^ Health and Social Research Center, Universidad de Castilla-La Mancha, Cuenca, Spain; ^3^ Investigación en Cuidados de la Salud Cardiovascular (CARVASCARE), Centro de Estudio Sociosanitarios, Universidad de Castilla-La Mancha, Cuenca, Spain; ^4^ Facultad de Ciencias de la Salud, Universidad Autónoma de Chile, Talca, Chile; ^5^ Network for Research on Chronicity, Primary Care, and Health Promotion (RICAPPS), Cuenca, Spain; ^6^ Facultad de Fisioterapia y Enfermería, Universidad de Castilla-La Mancha, Toledo, Spain; ^7^ Complejo Hospitalario Universitario de Albacete, Albacete, Spain

**Keywords:** renal transplant, musculoskeletal, metabolic, proportion, meta-analysis

## Abstract

**Introduction:**

Musculoskeletal disorders could be associated with metabolic disorders that are common after kidney transplantation, which could reduce the quality of life of patients. The aim of this study was to assess the prevalence of both musculoskeletal and metabolic disorders in kidney transplant patients.

**Methods:**

MEDLINE, CINAHL, Cochrane Library, EMBASE and Web of Science were searched from their inception up to June 2023. DerSimonian and Laird random-effects method was used to calculate pooled prevalence estimates and their 95% confidence intervals (CIs).

**Results:**

21,879 kidney transplant recipients from 38 studies were analysed. The overall proportion of kidney transplant patients with musculoskeletal disorders was 27.2% (95% CI: 18.4–36.0), with low muscle strength (64.5%; 95% CI: 43.1–81.3) being the most common disorder. Otherwise, the overall proportion of kidney transplant patients with metabolic disorders was 37.6% (95% CI: 21.9–53.2), with hypovitaminosis D (81.8%; 95% CI: 67.2–90.8) being the most prevalent disorder.

**Conclusion:**

The most common musculoskeletal disorders were low muscle strength, femoral osteopenia, and low muscle mass. Hypovitaminosis D, hyperparathyroidism, and hyperuricemia were also the most common metabolic disorders. These disorders could be associated with poorer quality of life in kidney transplant recipients.

**Systematic Review Registration:**

https://www.crd.york.ac.uk/prospero/, identifier [CRD42023449171].

## Graphical Abstract

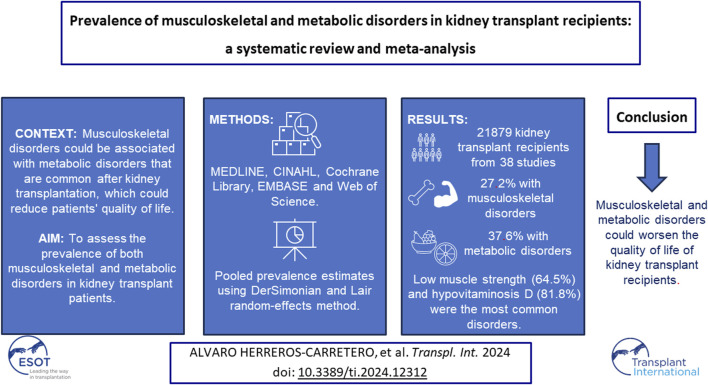

## Introduction

Renal transplantation represents the best therapy for patients diagnosed with end-stage renal disease. Major advances in surgical techniques and immunosuppressive treatment have led to a substantial improvement in the survival of these patients over the last few decades, resulting in a higher quality of life and lower treatment-related costs compared to dialysis [1, 2]. This surgical procedure involves the replacement of a healthy kidney, either from a living or deceased donor, in a patient whose kidneys are not functioning properly [1]. According to the Global Observatory on Donation and Transplantation (GODT3), a total of 65,668 kidney transplants were performed worldwide in 2021, making the kidney commonly the most transplanted organ [3].

Despite the improvement in the patient’s clinical status compared to the patient’s previous disease status, this therapy does not imply a cure [4]. The evolution of kidney transplant recipients will depend fundamentally on the use of immunosuppressive drugs, the origin of the transplanted kidney, the characteristics of the patient and several events that may occur in the post-transplant period [1], which pose certain risks to the health and quality of life of the transplant recipient. These post-transplant events include renal, infectious, urological, surgical, cardiovascular, and cerebrovascular complications, side effects of the drugs used to prevent rejection, and metabolic disorders [1].

In relation to the above, there are several metabolic disorders, such as hypercalcemia, hypophosphatemia, hyperparathyroidism, and hypovitaminosis D, among others, which are common in these patients and have the potential to cause loss of bone mineral density (BMD), as occurs with the use of glucocorticoids, whose doses are higher immediately after transplantation [2, 4, 5]. This loss of BMD leads to several musculoskeletal disorders that can affect the quality of life of transplant patients and need to be controlled.

Musculoskeletal disorders include a group of pathologies suffered by many patients after surgery, the exact prevalence of which is not yet well known [6]. This group includes disorders such as osteopenia, osteoporosis, and sarcopenia, which involve both a reduction in bone density and a reduction in strength and muscle mass, respectively [7]. Although it is a common complication in these patients, involving the loss of bone and muscle mass, especially in the first months after transplantation, both diagnosis and treatment to prevent these pathologies are still inadequate [8, 9]. Furthermore, there is a lack of studies that accurately synthesize and estimate the proportion of musculoskeletal and metabolic disorders in renal transplant patients. Therefore, the aim of this study was to carry out a systematic review and meta-analysis to determine the prevalence of musculoskeletal disorders and their related metabolic disorders in kidney transplant patients.

## Methods

This systematic review adhered to the Cochrane Collaboration Handbook, the Meta-analyses of Observational Studies in Epidemiology (MOOSE) guidelines, and the “Preferred Reporting Items for Systematic Reviews and Meta-Analysis” (PRISMA) guidelines [10]. This study was registered in the International Prospective Register of Systematic Reviews (PROSPERO) with the registration number (CRD42023449171).

### Search Strategy

A systematic search in MEDLINE (via PubMed), CINAHL, Cochrane Library, EMBASE (via Scopus), and Web of Science (WOS) was conducted from inception to June 2023. Gray literature and the references of selected studies were also reviewed to identify additional studies. The search strategy combined the following terms using Boolean operators: “post-kidney transplant,” “post-renal transplant,” “kidney transplant,” “renal transplant,” “musculoskeletal,” “muscular pain,” “muscle pain,” sarcopenia, fibromyalgia, myopathy, “joint pain,” fracture, fragility, “bone pain syndrome,” “bone syndrome,” “bone pain,” “bone disease,” “bone disorder,” “lower limb pain,” hyperparathyroidism, hypophosphatemia, gout, hyperuricemia, arthritis, “bone loss,” osteoporosis, osteopenia, osteomalacia, “mineral disorder,” hypercalcemia, “vitamin D,” “hypovitaminosis D,” “vitamin D deficiency.” The references of the included studies were also checked. If the full text of a study was not available, the authors of the study were contacted. The systematic search was conducted independently by two investigators (AH-C and MG-M). The detailed search strategy is available in Supplementary Table S1.

### Eligibility Criteria

Observational studies analysing musculoskeletal and metabolic disorders developed in kidney transplant patients were included. The inclusion criteria were as follows: 1) population: adult patients over 18 years of age; 2) study design: cross-sectional or baseline data from longitudinal studies without language restriction; and 3) outcome: primary outcomes including prevalence of musculoskeletal or metabolic disorders in kidney transplant recipients. Exclusion criteria were as follows: 1) ineligible publication types (clinical trials, literature reviews, commentaries, or letters to the editor); 2) patients with other previous nonrenal transplants; 3) pregnant or breastfeeding women; and 4) no access to full text.

### Data Extraction

After selecting the studies that met the inclusion criteria, the following data were collected and described in a descriptive table (Table 1): (a) first author and year of publication; (b) country; (c) study design; and (d) sample characteristics (year of transplantation, number of participants, age, and sex); and (e) outcome analysed. If more than one study provided data on the same sample, the study with the most detailed results and/or with the largest sample size was selected for data synthesis.

**TABLE 1:** Characteristics of the studies included (*n* = 38).

Author and year	Country	Study design	Sample characteristics					Outcome (prevalence)
			Transplant year	n	Age and gender (% women)	Time since transplant	Time on haemodialysis prior to transplant	
Alagoz S. et al. [11] 2019	Turkey	Retrospective longitudinal	2002–2012	176	32.9 ± 11.8 (38.1)	1 month	33.8 ± 33.1 months	Hypercalcemia (18.2%) Hypophosphatemia (33.3%) Hyperparathyroidism (45.3%)
						12 months		Hypercalcemia (17.2%) Hypophosphatemia (8.6%) Hyperparathyroidism (29.4%)
						60 months		Hypercalcemia (13.2%) Hypophosphatemia (11.4%) Hyperparathyroidism (9.2%)
Amin T. et al. [12] 2016	Australia	Cross-sectional	1971–2011	679	55 ± 13 (39)	≥3 months	28.8 ± 24 months	Hypercalcemia (15%)
Batteux B. et al. [13] 2020	France	Prospective longitudinal	2012–2018	310	51.1 ± 12.8 (37.4)	1 month	30 months	Osteopenia: lumbar area (34.5%); femoral area (53.5%)
								Osteoporosis: lumbar area (6.1%); femoral area (10%)
Berga JK. Et al [14]. 2010	Spain	Retrospective longitudinal	—	110	50.2 ± 11 (53)	—	—	Hypovitaminosis D (96.4%): insufficiency (43.6%), deficiency (52.7%)
Braga Jr JWR. et al. [15] 2006	Brazil	Cross-sectional	2000	191	44.8 ± 0.8 (50.8)	87 ± 3.7 months	46.48 ± 3.03 months	Osteopenia: lumbar area (32.5%); femoral area (33%)
								Osteoporosis: lumbar area (11.5%); femoral area (11%)
								Fractures (24.1%)
Chan W. et al. [16] 2019	United Kingdom	Prospective longitudinal	2010–2013	128	49 ± 15 (44)	60 (12–132) months	—	Sarcopenia (28.9%)
								Low muscle strength (64.1%)
								Low muscle mass (35.9%)
Conley E. et al. [17] 2008	United States	Retrospective longitudinal	1998–2006	554	46.3 ± 0.5 (42.2)	14 months	—	Fractures (13%)
Einollahi E. et al. [18] 2013	Iran	Cross-sectional	2008–2011	4,217	38 ± 15 (36)	60 months	—	Hyperuricemia (31.8%)
Evenepoel P. et al. [19] 2019	Belgium	Cross-sectional	2006–2013	518	54.7 ± 12.8 (39.4)	>2 weeks	—	Hypovitaminosis (38.4%): insufficiency (35.1%); deficiency (3.3%)
								Osteopenia: lumbar area (8.1%); femoral area (55%)
								Osteoporosis: lumbar area (23.7%); femoral area (22%)
								Fractures (7.3%)
Férnandez Castillo R. et al. [20] 2018	Spain	Cross-sectional	—	119	−(41.2)	6 months	—	Osteopenia: lumbar area (32.9%); femoral area (49.3%)
								Osteoporosis: lumbar area (30.1%); femoral area (15.1%)
						12 months		Osteopenia: lumbar area (38.4%); femoral area (51.5%)
								Osteoporosis: lumbar area (30.8%); femoral area (16.7%)
Gregorini M. et al. [21] 2017	Italy	Cross-sectional	2000–2016	297	55.5 ± 12 (34.7)	24 months	—	Osteopenia: lumbar area (40.4%); femoral area (50.2%)
								Osteoporosis: lumbar area (13.8%); femoral area (20.9%)
								Fractures (12.1%)
Hamidian Jahromi A. et al. [22] 2009	England	Prospective longitudinal	2000–2002	121	35.5 ± 12.5 (30.6)	3 months	17.4 ± 6 months	Hypercalcemia (17.4%)
								Hyperparathyroidism (9.9%)
						12 months		Hypercalcemia (5.7%)
								Hyperparathyroidism (5.7%)
Jerman A. et al. [23] 2017	Slovenia	Cross-sectional	1976–2011	507	54.3 ± 12 (45)	116.4 months	63.4 ± 43.6 months	Fractures (12.6%)
Jørgensen HS. et al. [24] 2016	Norway	Cross-sectional	2006–2011	701	52.2 ± 14.7 (32.4)	2.5 months	13.8 (7.8–26.3) months	Osteopenia: lumbar area (35.7%); femoral area (51.8%)
								Osteoporosis: lumbar area (16.8%); femoral area (26%)
Khosravi M. et al. [25] 2020	Iran	Cross-sectional	—	148	43.8 ± 12.7 (48)	67.59 ± 42.66 months	14.18 ± 16.05 months	Osteopenia: lumbar area (49.3%)
								Osteoporosis: lumbar area (18.9%)
Kim KM. et al. [26] 2010	South Korea	Cross-sectional	1990–2008	356	39.3 ± 10.3 (39.3)	102.63 ± 27.25 months	—	Hyperuricemia (15.4%)
Kosoku A. et al. [27] 2020	Japan	Cross-sectional	—	210	55 ± 10 (42)	85 (43–135) months	19 (6–67) months	Sarcopenia (11%)
Limirio LS. et al. [28] 2019	Brazil	Cross-sectional	—	127	47.6 ± 11.5 (31.5)	95.5 ± 78.2 months	55.4 ± 43.5 months	Sarcopenia (50.4%)
								Low muscle strength (80.3%)
								Low muscle mass (61.4%)
López Ruiz ML. et al. [29] 2015	Spain	Cross-sectional	2002–2009	306	46.9 ± 13.8 (37.6)	12 months	—	Osteopenia: lumbar area (14.4%); femoral area (19.6%)
								Osteoporosis: lumbar area (12.4%); femoral area (6.9%)
Malheiro J. et al. [30] 2012	Portugal	Cross-sectional	1983–2010	302	49.6 ± 13.4 (39.4)	91.2 (27.6–170.4) months	—	Hyperuricemia (42.1%)
Marcén R. et al. [31] 2009	Spain	Cross-sectional	—	509	45.4 ± 14.5 (42)	113 ± 76 months	—	Hypovitaminosis D (85.3%): insufficiency (47%); deficiency (38.3%)
Menna Barreto APM. et al. [32] 2019	Brazil	Cross-sectional	—	185	50 ± 7 (43)	117 (32–173) months	—	Sarcopenia (17.3%)
								Low muscle strength (45.9%)
								Low muscle mass (23.8%)
Muirhead N. et al. [33] 2014	Canada	Retrospective longitudinal	2003–2008	1,000	50 ± 12.5 (35.6)	12 months	—	Hypercalcemia (16.6%)
								Hyperparathyroidism (47.6%)
						24 months		Hypercalcemia (13.6%)
								Hyperparathyroidism (51.1%)
						36 months		Hypercalcemia (9.5%)
								Hyperparathyroidism (43.4%)
						48 months		Hypercalcemia (10.1%)
								Hyperparathyroidism (39.3%)
Ozkayar N. et al. [34] 2014	Turkey	Cross-sectional	—	166	37.9 ± 11.9 (41)	—	—	Sarcopenia (20.5%)
Park WY. et al. [35] 2017	United Kingdom	Prospective longitudinal	2011–2013	207	45 ± 11 (46.4)	12 months	25.3 months	Osteopenia: femoral area (40.1%)
								Osteoporosis: femoral area (47.3%)
Patel S. et al. [36] 2001	United Kingdom	Cross-sectional	1998	165	46 ± (42)	61.2 months	18 months	Osteopenia: lumbar area (30.9%); femoral area (40.6%)
								Osteoporosis: lumbar area (7.9%); femoral area (10.3%)
								Fractures (16.4%)
Savaj S. et al. [37] 2012	Iran	Cross-sectional	2010	113	46.1 ± 13.6 (51.3)	106.4 ± 77.0 months	147.1 ± 92.8 months	Hyperparathyroidism (76.1%)
								Hypovitaminosis D (94.7%): insufficiency (49.6%). deficiency (45.1%)
								Osteopenia: lumbar area (52.2%); femoral area (36.3%)
								Osteoporosis: lumbar area (12.4%); femoral area (45.1%)
Schreiber W. et al. [38] 2020	Switzerland	Prospective longitudinal	2008–2009	135	51 ± 11 (33.3)	6 months	—	Vitamin D deficiency (65.2%)
Segaud N. et al. [39] 2018	France	Prospective longitudinal	2005–2011	259	49.7 ± 12.1 (37.1)	8.8 ± 1.9 months	38.4 months	Osteopenia: femoral area (42.9%)
								Osteoporosis: femoral area (40.9%)
								Fractures (10.8%)
Simbolon FR. et al. [40] 2018	Taiwan	Retrospective longitudinal	1997–2010	5,917	45.1 ± 11.9 (48.6)	32.4 months	—	Gout (8.8%)
Stamp L. et al. [41] 2006	New Zealand	Cross-sectional	2004	202	53 (31.9)	>36 months	—	Gout (23.3%)
Torres A. et al. [42] 2016	Spain	Cross-sectional	2008–2010	727	55 ± 13.6 (39.9)	>12 months	67 ± 29 months	Hypercalcemia (6.5%)
								Hypophosphatemia (6.2%)
								Hyperparathyroidism (76.9%)
								Hypovitaminosis D (83.2%): insufficiency (50.8%); deficiency (32.5%)
								Fractures (14.6%)
Velioglu A. et al. [43] 2021	Turkey	Cross-sectional	2017–2018	153	46.5 ± 11.9 (50.3)	86.4 months	35 months	Hyperparathyroidism (52.9%)
								Hypovitaminosis D (68.8%): insufficiency (49.7%); deficiency (19%)
								Osteopenia: lumbar area (28.1%); femoral area (41.2%)
								Osteoporosis: lumbar area (7.2%); femoral area (7.8%)
								Fractures (43.4%)
Vilarta CF. Et al [44]. 2017	Brazil	Cross-sectional	—	149	44 ± −(56.4)	72 months	—	Hypovitaminosis D (79.2%): insufficiency (37.6%); deficiency (41.6%)
								Fractures (10%)
Wang C. et al. [45] 2021	China	Cross-sectional	—	216	41.5 ± 9.9 (27.8)	—	15 months	Hypercalcemia (8.8%)
								Hypophosphatemia (3.7%)
								Hypovitaminosis D (78.7%): insufficiency (46.3%); deficiency (32.4%)
								Fractures (3.2%)
Weng SC. et al. [46] 2014	Taiwan	Prospective longitudinal	1999–2013	880	48.7 ± 12.3 (46.8)	—	—	Hyperuricemia (44.2%)
								Gout (17.7%)
Wolf M. et al. [47] 2016	United States	Prospective longitudinal	—	246	52.8 ± 13.4 (36.7)	—	42 ± 34.8 months	-Hypercalcemia (30.5%)
								-Hypophosphatemia (53.7%)
								-Hyperparathyroidism (89.4%)
Zhang K. et al. [48] 2015	China	Retrospective longitudinal	2008–2011	573	41.4 ± 9.5 (31.6)	1 month	—	-Hyperuricemia (16.2%)
						3 months		-Hyperuricemia (24.1%)
						24 months		-Hyperuricemia (30.9%)
						36 months		-Hyperuricemia (42.8%)

Two reviewers (AH-C and MG-M) independently conducted the data extraction, and disagreements were resolved by consensus or by a third reviewer (CB-M). Articles retrieved were imported and managed by Mendeley reference manager.

### Methodological Quality Assessment

To assess the methodological quality of the studies included in this systematic review and meta-analysis, we used the Joanna Briggs Institute (JBI) tool *“Checklist for prevalence studies”* scale by Munn et al [49] for cross-sectional descriptive studies and the JBI tool *“Checklist for cohort studies”* scale by Moola et al [50] for longitudinal cohort studies. Both scales [49, 50] consist of 9 and 11 items, respectively. They are scored as “yes” (1), “no” (0), “not applicable” (NA) and “unclear” (?). The final score for each study therefore ranged from 0 to 9 or 11. Depending on this score, each study was classified as having a low (>7), moderate (4–6) or high (1–3) risk of bias [49, 50].

Both the data extraction and the quality assessment were performed independently by two reviewers (AH-C and MG-M), and disagreements were resolved by consensus or by involving a third reviewer (CB-M).

### Statistical Analysis and Data Synthesis

Pooled prevalence estimates with their respective 95% confidence intervals (CIs) were calculated for each subgroup of musculoskeletal disorders (sarcopenia, low muscle strength, and low muscle mass, osteopenia, osteoporosis, fractures, and gout) and metabolic disorders subgroup (hypercalcemia, hypophosphatemia, hyperparathyroidism, hyperuricemia, and hypovitaminosis D). In addition, the overall pooled prevalence of both musculoskeletal and metabolic disorders was also estimated. DerSimonian and Laird random-effects method [51, 52] was used to calculate pooled prevalence estimates and their 95% CIs. Heterogeneity between studies was assessed using the *I* ^2^ statistic [53] with values considered as follows: not important (0%–40%), moderate (30%–60%), substantial (50%–90%) and considerable heterogeneity (75%–100%). The significance value of the pooled effect size was estimated based on the 95% CI. Two-sided *p* values of .05 or less were considered significant.

We conducted a sensitivity analysis to determine the robustness of the summary estimates by removing each included study from the analysis one by one. Furthermore, meta-regression models were performed considering mean age, percentage of women, time on hemodialysis prior to transplant, and time since transplant to determine their influence on prevalence estimates. Due to the limited number of studies included (*n* < 10) in each subgroup analysis, meta-regression analysis was only performed with the following outcome variables: osteopenia (lumbar area), osteopenia (femoral area), osteoporosis (lumbar area), osteoporosis (femoral area) and fractures.

Statistical analyses were performed using Stata SE software, version 15 (StataCorp) and Comprehensive Meta-Analysis V3. Global prevalence was estimated using the STATA metaprop statistical package.

## Results

### Study Selection

**FIGURE 1:**
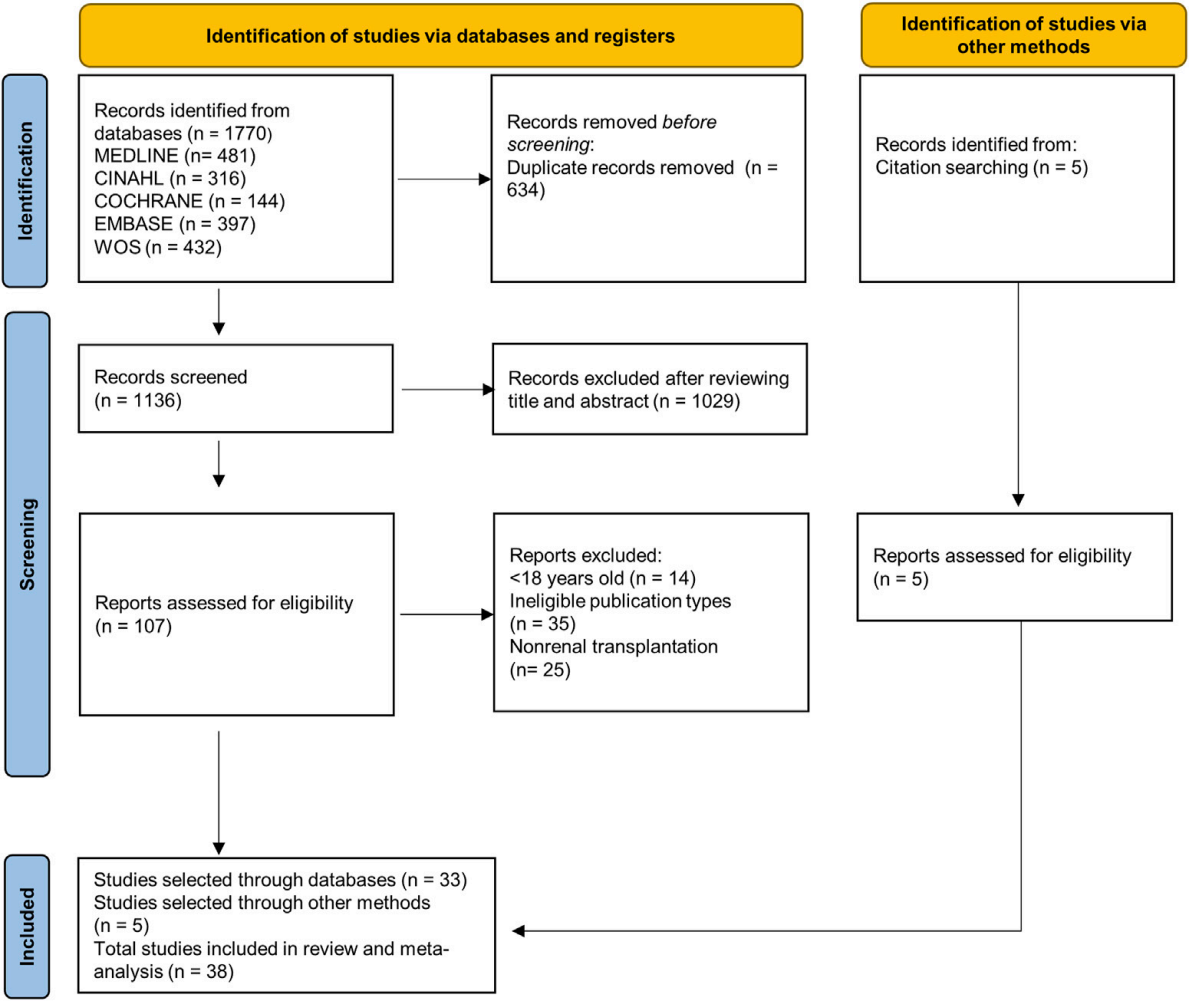
PRISMA 2020 flowchart.

A total of 1,770 articles were retrieved from the bibliographic search. After removing duplicates, a total of 38 articles [11–48] were selected for quantitative synthesis (Figure 1).

#### Characteristics of the Selected Studies

The characteristics of the studies selected for this systematic review and meta-analysis are detailed in Table 1. The study design was cross-sectional in 27 studies [11, 12, 14, 15, 18–21, 23–32, 34–37, 41–45] (71.1%) and longitudinal in 11 [13, 16, 17, 22, 33, 38–40, 46–48] (28.9%). All these articles were published between 2001 and 2021, and most of them were conducted in Europe [13, 14, 16, 19–24, 29–31, 35, 36, 38, 39, 42] (44.7%), although there were also studies from Asia [11, 18, 25–27, 34, 37, 40, 43, 45, 46, 48] (31.6%), America [15, 17, 28, 32, 33, 44, 47] (18.4%) and Oceania [12, 41] (5.3%).

A total of 21,879 patients (41.4% women) with a mean age of 45.4 years were analysed in this study. The kidney transplants were performed between 1971 and 2018. The mean time since transplantation was 41.3 months (3.4 years), and the mean time on dialysis before transplantation was 36.08 months (3 years).

The musculoskeletal disorders analysed in this review were sarcopenia, low muscle strength and low muscle mass, osteopenia and osteoporosis, bone fractures and gout. The outcomes analysed for metabolic disorders were hypercalcemia, hypophosphatemia, hyperparathyroidism, hyperuricemia and hypovitaminosis D.

#### Study Quality

Of the cross-sectional studies (Supplementary Table S2), 96.3% and 3.7% had a low and a moderate risk of bias, respectively. For longitudinal studies, 54.5% and 45.6% had a low and a moderate risk of bias, respectively (Supplementary Table S3). Considering all the studies, 84.2% and 15.8% had a low and moderate risk of bias, respectively.

#### Main Results

A general estimate of the outcomes regarding both musculoskeletal and metabolic disorders is shown in Figures 2, 3, respectively. Each outcome was also independently analysed and is shown in Supplementary Figures S1–S16. The overall proportion of kidney transplant patients with musculoskeletal disorders was 27.2 (95% CI: 18.4–36.0; *I* ^2^ = 92.3%) (Figure 2), and that with metabolic disorders was 37.6% (95% CI: 21.9–53.2; *I* ^2^ = 97.8%) (Figure 3).

(i) Musculoskeletal disorders
  (a) Muscle disorders (sarcopenia, low muscle strength and low muscle mass): The prevalence of sarcopenia was analysed in five studies [16, 27, 28, 32, 34]. A total of 816 individuals were included, with an overall prevalence of 23.6% (95% CI: 13.2–38.5; *I* ^2^ = 94.1) (Supplementary Figure S1). Three studies [16, 28, 32], with 440 subjects, included the other two outcomes. For low muscle strength, the overall prevalence was 64.5% (95% CI: 43.1–81.3; *I* ^2^ = 94.4), and the prevalence of low muscle mass was 39.5% (95% CI: 20.3–62.6; *I* ^2^ = 95.3) (Supplementary Figures S2, S3).
  (b) Osteopenia and osteoporosis: Eleven articles [13, 15, 19–21, 24, 25, 29, 36, 37, 43] investigated the prevalence in the lumbar area, and 12 [13, 15, 19–21, 24, 29, 35–37, 39, 43] studied the prevalence in the femoral area, with 3,021 and 3,339 transplant recipients, respectively. The prevalence of osteopenia in the lumbar area was 30.7% (95% CI: 23.3–39.3; *I* ^2^ = 95.1). In the femoral area, it was 42.6% (95% CI: 36.5–48.8; *I* ^2^ = 91.9) (Supplementary Figures S4, S5). For lumbar osteoporosis, the prevalence was 13.8% (95% CI: 10.4–17.9; *I* ^2^ = 88.2). Finally, for the femoral area, the prevalence was 19.2% (95% CI: 13.4–26.7; *I* ^2^ = 95.6) (Supplementary Figures S6, S7).
  (c) Fractures: Eleven articles [15, 17, 19, 21, 23, 36, 39, 42–45] assessed this outcome. The prevalence in 3,736 patients was 13.1% (95% CI: 9.6–18.5; *I* ^2^ = 93.1) (Supplementary Figure S8).
  (d) Gout: Three studies [40, 41, 46] analysed the prevalence of this disorder in renal transplant recipients, including 6,999 participants, where the overall prevalence of gout was 15.4% (95% CI: 8.3–26.9; *I* ^2^ = 97.3) (Supplementary Figure S9).
(ii) Metabolic disturbances.
  (a) Hypercalcaemia: Seven studies [11, 12, 22, 33, 42, 45, 47] provided data on this disorder, with a total of 3,165 subjects analysed. The overall prevalence in this population was 15.7% (95% CI: 14.5–17.0; *I* ^2^ = 91.3) (Supplementary Figure S10).
  (b) Hypophosphatemia: Four studies [11, 42, 45, 47] analysed the prevalence of hypophosphatemia among kidney transplant recipients. The overall prevalence of 1,365 individuals was 12.4% (95% CI: 4.3–31.2; *I* ^2^ = 98.1) (Supplementary Figure S11).
  (c) Hyperparathyroidism: The prevalence of this disorder was obtained from seven studies [11, 22, 33, 37, 42, 43, 47]. The overall prevalence obtained in this population of 2,536 subjects was 47.6% (95% CI: 31.3–64.5; *I* ^2^ = 98.2) (Supplementary Figure S12).
  (d) Hyperuricemia: Hyperuricemia was analysed in five studies [18, 26, 30, 46, 48], with a total of 6,328 subjects, with metabolic disorders having the largest population. The overall prevalence was 29.8% (95% CI: 23.8–36.7; *I* ^2^ = 96.8) (Supplementary Figure S13).
  (e) Hypovitaminosis D: Eight studies [14, 19, 31, 37, 42–45] analysed the prevalence of this disorder. In this population of 2,495 people, the overall prevalence was 81.8% (95% CI: 67.2–90.8; *I* ^2^ = 98.2), with this metabolic disorder being the most common finding (Supplementary Figure S14). This alteration was divided into vitamin D insufficiency and vitamin D deficiency. For the former, the prevalence was 44.9% (95% CI: 40.1–49.7; *I* ^2^ = 80.7), and for vitamin D deficiency, it was 32.9% (95% CI: 23.1–44.4; *I* ^2^ = 96.3) (Supplementary Figures S15, S16).

**FIGURE 2:**
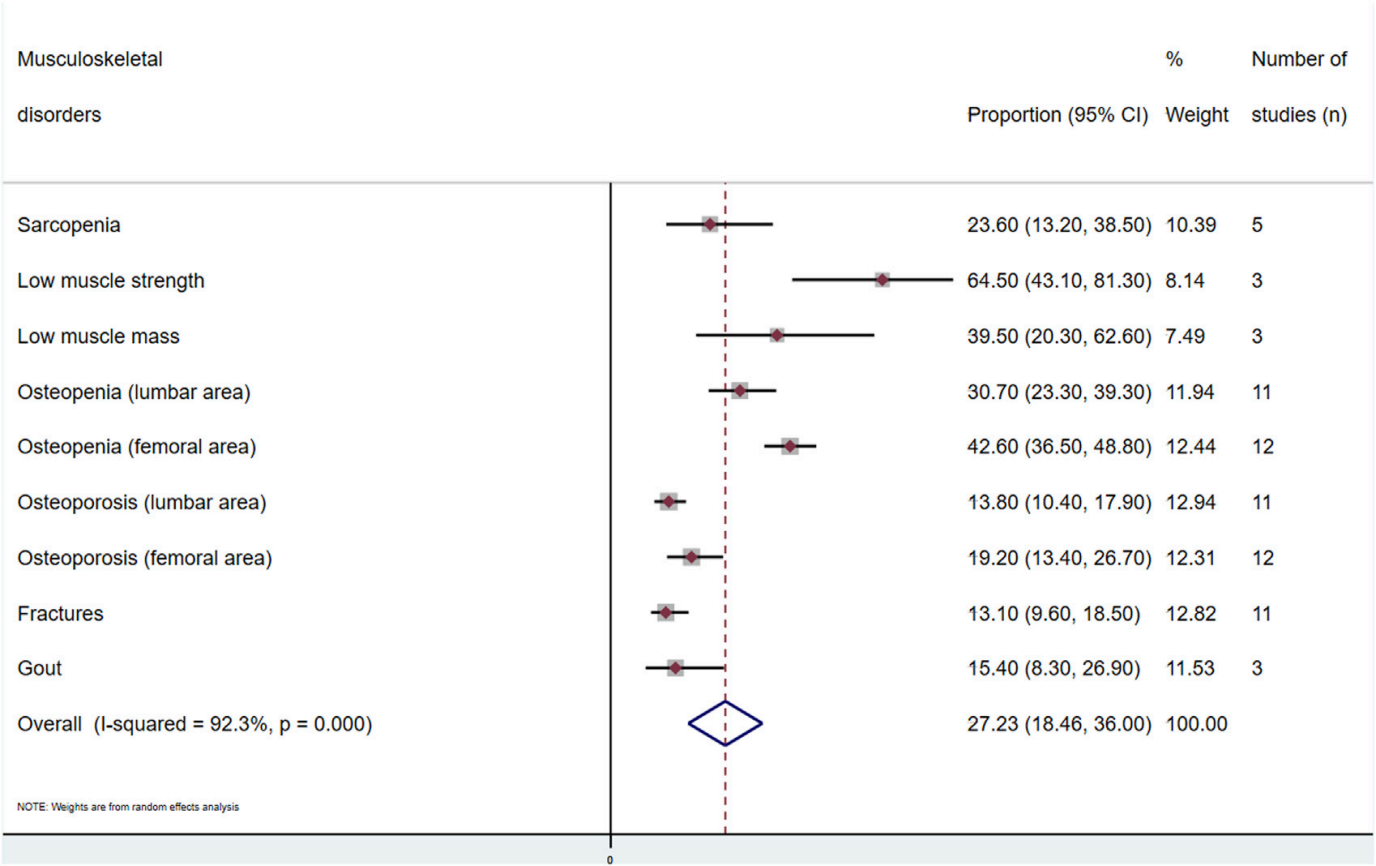
Meta-analysis of the proportion of musculoskeletal disorders in kidney transplant recipients.

**FIGURE 3:**
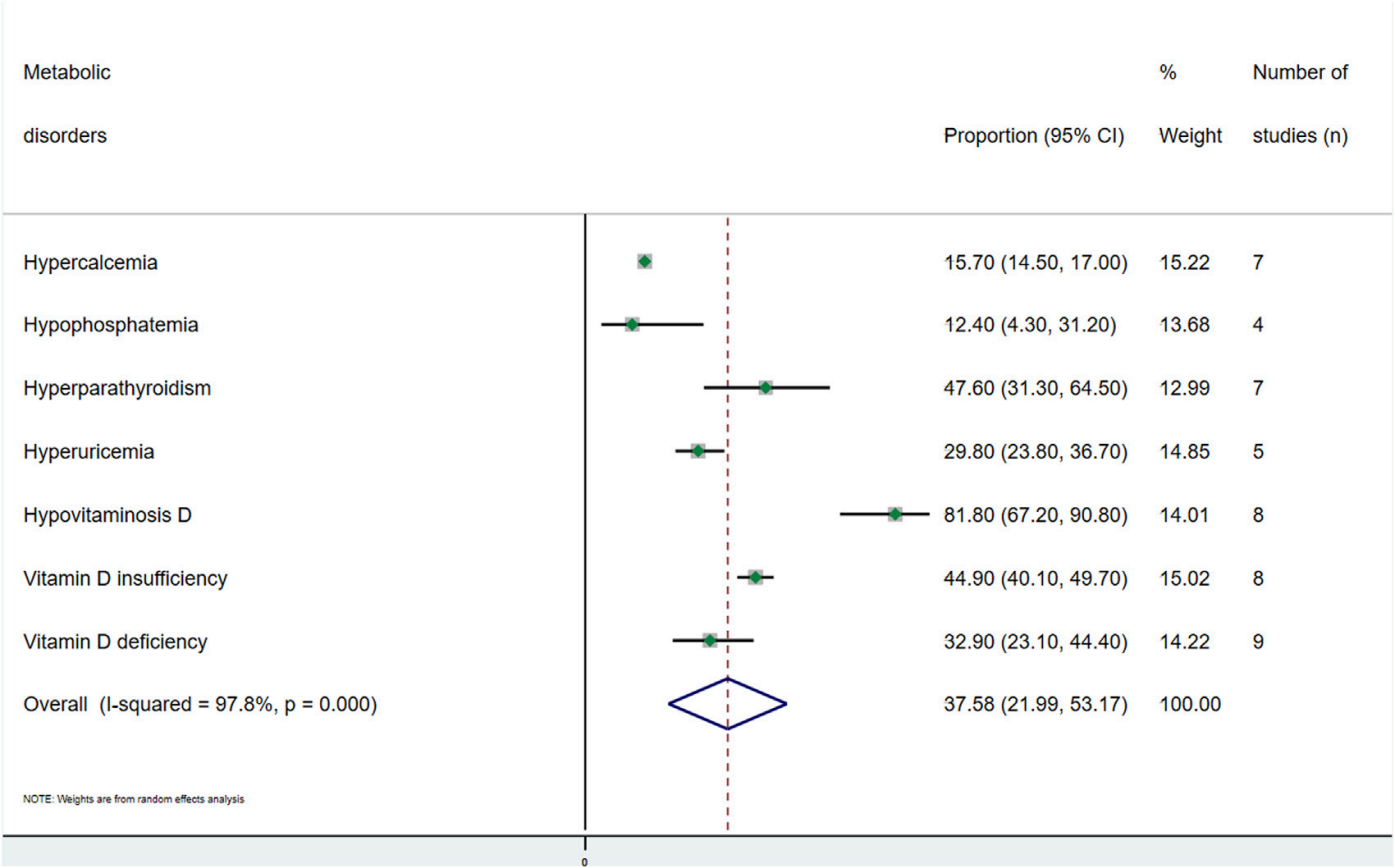
Meta-analysis of the proportion of metabolic disorders in kidney transplant recipients.

These results obtained have been compared with the results of other studies [54–61] that analyse the same variables in the general population (who have not received a kidney transplant). Among metabolic disturbances, the comparison is as follows: 14.9% vs. 0.8% (general population) for hypercalcemia, 58.0% vs. 0.8% for hyperparathyroidism, 31.2% vs. 13.3% for hyperuricemia, and 81.8% vs. 15.7% for hypovitaminosis D. Regarding musculoskeletal disorders, the differences are as follows: 23.6% vs. 15.5% for sarcopenia, 39.5% vs. 27.0% for low muscle mass, 30.7/42.6% (lumbar/femoral area) vs. 40.4% for osteopenia, 13.8/19.2% (lumbar/femoral area) vs. 18.3% for osteoporosis, 14.2% vs. 1.1% for fractures, and 15.4% vs. 1.1% for gout. This comparison is shown in detail in Supplementary Tables S4, S5, and individually the comparison of each variable can be seen in Supplementary Figures S17–S26.

### Sensitivity and Meta-Regression Analysis

When the impact of individual studies was examined by removing studies from the analysis one by one, the estimate of the proportion of sarcopenia changed after removing the Limirio LS. sample [28] (from 23.6% to 19.1%); Menna Barreto ANP. [32] for low muscle strength (from 64.5% to 71.4%); Limirio LS. [28] for low muscle mass (from 39.5% to 29.1%); Wang C. [45] for fractures (from 13.10% to 16.0%), and Simbolon FR. [40] for gout (from 15.4% to 19.9%) (Supplementary Table S6). On the other hand, regarding the estimated proportions of metabolic disorders, these were modified after removing the samples of Wolf M. [47] for hypercalcemia (from 15.7% to 13.0%) and hypophosphatemia (from 12.4% to 7.7%), and Evenepoel P. [19] for vitamin D deficiency (from 32.9% to 40.0%) (Supplementary Table S7).

Meta-regression models showed that all the variables considered (age, %females, time since transplant and time on haemodialysis prior to transplant) influenced the prevalence estimates of the outcome variables analysed (Supplementary Table S8).

## Discussion

The main objective of this systematic review and meta-analysis was to provide a complete synthesis of the prevalence of musculoskeletal disorders and metabolic disorders in kidney transplant patients. There is a wide range in the prevalence of metabolic disturbances and musculoskeletal disorders in this population. The most common metabolic disorders in this group of patients were hypovitaminosis D (81.8%), hyperparathyroidism (47.6%), and hyperuricemia (29.8%). Among the musculoskeletal disorders, the most common were low muscle strength (64.5%), femoral osteopenia (42.7%), and low muscle mass (39.5%).

Renal transplantation solves many problems of end-stage renal disease; however, certain metabolic disturbances may persist for some time. Hyperparathyroidism, hypercalcemia, hypophosphatemia, hypovitaminosis D, and hyperuricemia are common after transplantation, and they often occur simultaneously. In addition, together with other factors, they may be involved in the development of certain musculoskeletal disorders that affect the quality of life of the transplanted patient [62].

Despite the improvement in renal function after transplantation, hyperparathyroidism may develop due to a number of factors, including the high levels of parathyroid hormone (PTH) prior to transplantation, the prolonged period of renal disease and dialysis, the degree of hyperplasia of the parathyroid gland or the decrease in vitamin D [2, 62–64]. PTH levels begin to decline during the first 3–6 months after the procedure, but according to the article published by Hassan et al [6], high PTH levels can still be found in 30%–60% of patients 1 year after transplantation [6]. This alteration is also associated with hypercalcemia and hypophosphatemia, among others, which could lead to loss of BMD [2].

Approximately 15% of patients with hyperparathyroidism also have hypercalcemia [65]. PTH increases blood calcium levels by transporting calcium from the bones into the blood, facilitating calcium reabsorption in the kidneys and its absorption in the digestive system [62]. However, it is not the only factor that allows an increase in blood calcium. The increase in vitamin D levels after transplantation also increases calcium absorption in the intestine, as well as the bone resorption that can occur after the procedure [5, 62]. It is important to emphasize that hypercalcaemia is not a cause of BMD loss but rather a consequence [62]. This alteration is reported in 5%–15% of the transplanted population after the intervention, according to the article published by Bouquegneau et al [9], and is more common at 3–6 months, especially in patients with higher blood PTH levels [9]. This change may resolve in some patients 6–8 months after transplantation, but in others, it may take years [64]. Hypercalcemia may play a role in triggering nephrolithiasis, rejection, and dysfunction of the transplanted kidney [65].

Another change associated with hyperparathyroidism is hypophosphatemia. Like calcium, PTH is involved in the regulation of phosphorus in the body [62]. In addition, there is another hormone in the body called fibroblast growth factor 23 (FGF-23), which has been identified as the main phosphorus-regulating factor in the body. This hormone is secreted by bone cells, and its production is partially stimulated by PTH. It has also been described that excess FGF-23 [66] is produced in bone mineralization disorders. These two hormones contribute to a decrease in the reabsorption of phosphorus in the kidney, which is why kidney transplant recipients have low levels of this metabolite [64]. This alteration is common during the first 3 months after transplantation, and according to the study by Bouquegneau et al [9], it occurs in 50% of transplant recipients and stabilizes after 6–12 months [9, 67]. This alteration is associated with a decrease in osteoblast activity, resulting in deficient bone mineralization [9].

Vitamin D metabolism is also affected after renal transplantation. Hypovitaminosis D is associated with immunosuppressive therapy, residual renal function after transplantation, malabsorption, poor diet or reduced exposure to sunlight [62, 64]. The vitamin D status of the body is tested by blood levels of calcidiol, a precursor of this vitamin; hypovitaminosis D is therefore understood to be a blood level of calcidiol of less than 30 ng/mL, with vitamin D insufficiency being between 15 and 30 ng/mL and vitamin D deficiency being less than 15 ng/mL [44, 68]. According to the article published by Evenepoel et al, [68] the prevalence of hypovitaminosis D in the third month after renal transplantation is 78%, and according to the articles by Bouquegneau et al [9] and Alshayeb et al, [64] vitamin D deficiency would be present in 30% of the operated patients. As renal function recovers, vitamin D levels begin to rise, although they remain lower than those of the general population (Supplementary Table S4) [8, 62]. Hypovitaminosis D may be associated with lower transplant tolerance, worsening infections, and an upset in BMD [5].

This BMD alteration is associated with both osteopenia and osteoporosis, as mentioned above, with various metabolic disturbances that occur after renal transplantation [4]. Although there are also several risk factors, such as advanced age, sex, and ethnicity of the patient [4, 6], the main underlying factor for BMD loss is treatment with glucocorticoids after transplantation. Glucocorticoids inhibit bone tissue formation and increase osteoclast activity by decreasing the formation and differentiation of osteoblasts [69]. The difference between osteopenia and osteoporosis is the amount of BMD lost, with osteoporosis considered a more severe pathology than osteopenia. These disorders occur most frequently in the first 6–12 months after transplantation, with the greatest loss of BMD in the first 6 months [70]. In our meta-regression analysis, we have also shown a negative correlation between the time of transplantation and the prevalence of osteoporosis and osteopenia [20, 70]. After 6 months, this loss of bone mineral slows down, probably due to the decrease in glucocorticoid use and the gradual correction of the various metabolic disturbances associated with these disorders. According to the study published by Ebeling et al, [71] the presence of osteoporosis can be found in 17%–49% of kidney transplant recipients in the lumbar area and in 11%–56% of patients transplanted in the femoral area [71]. In our meta-analysis, the prevalence of osteoporosis in the lumbar area was 13.8%. In contrast, in the femoral area, the prevalence was 19.2%.

The main consequence of BMD loss is an increased risk of fractures [2], although there are several factors, such as advanced age, loss of muscle mass and reduced physical activity, that may increase the risk of this adverse event [4]. Approximately 22.5% of patients suffer at least one fracture in the first 5 years after transplantation, which means an incidence 4 times higher than that for the general population but lower than that in patients who remain on dialysis (Supplementary Table S5) [9]. Fractures are associated with increased hospitalization and mortality in kidney transplant recipients, with hip, ankle, and foot fractures being the most common, suggesting a large economic impact [9, 72, 73]. In our study, the prevalence of fractures after transplant was 14.2%.

Another factor that could increase the risk of fractures in transplant recipients is the loss of muscle mass and muscle strength associated with sarcopenia, which is associated with an increased risk of falls, physical disability, lower quality of life and greater morbidity and mortality [73]. The main risk factors for sarcopenia in kidney transplant recipients are vitamin D deficiency, physical inactivity, prolonged hospitalization, nutritional deficiencies, hyperparathyroidism, and proteinuria [7, 74]. The prevalence of this condition, whose occurrence in kidney transplant recipients is estimated at a younger age in comparison with the general population [75], varies greatly because there are no universal diagnostic criteria. In addition, together with osteopenia and osteoporosis, the risk of fracture in these patients increases considerably [7].

On the other hand, gout, which is a type of arthritis that occurs after the deposition of uric acid crystals in the joints, causing attacks of pain and inflammation, is another disorder that could be associated with kidney transplantation [76]. The main cause for this disorder is hyperuricemia, a metabolic disturbance that is common after renal transplantation. According to the article by Gupta et al, [77] hyperuricemia could reach a prevalence of 10%–84%, while gout could be present in 2%–28% of transplant recipients. In our study, the prevalence of gout was specifically 15.4%, although the differences in the date of transplantation between the three included studies may limit the generalisability of this estimate.

In order to compare these results, we have found a series of studies [54–61] that provide prevalence data for the variables analysed in the general population (Supplementary Tables S4, S5; Supplementary Figures S17–S26). It should be noted that for the variables of hypophosphatemia and low muscle strength, we have not been able to find any study providing prevalences in the general population. As for the other variables, we can observe the large difference in prevalence estimates between the transplanted population and the general population for hypercalcemia [54], hyperparathyroidism [55], hyperuricemia [55], hypervitaminosis D [56], sarcopenia [57], low muscle mass [58], fractures [59] and gout [55], indicating an increase in the prevalence of these metabolic disturbances and musculoskeletal disorders after kidney transplantation. In the case of osteopenia and osteoporosis, the difference in prevalence between the two populations is small, although it is true that in the studies [60, 61] we have found in the general population, it is not divided into zones, as it is in the studies [13, 15, 19–21, 24, 25, 29, 35–37, 39, 43] in the transplanted population, which divide the prevalence into lumbar and femoral areas, so that no conclusions could be drawn when comparing the two populations with regard to these two variables analysed.

This study has several potential limitations, and its findings should be interpreted with some caution. First, they are inherent to the conduct of a systematic review and meta-analysis (selection bias and limited information reported by original studies). Second, the design of most studies was retrospective and cross-sectional, which does not allow establishing a cause-effect relationship. Additionally, the heterogeneity of the results was high, which may limit the extrapolation of data to different populations. Thirdly, the results should be interpreted with caution, given the pooling of studies from different years and geographical locations, with different circumstances and sample characteristics. In this sense, studies were conducted in five different decades, in which the surgical techniques, metabolic goals, and available medications may have been different. On the other hand, there was variability in the time at which the different outcomes were measured after transplantation. Finally, despite the physiological link between hyperparathyroidism and hypercalcemia and hypophosphatemia, our results did not show a clear association between their prevalence estimates. The main reasons for this finding could be the coexistence of some lifestyle-related covariates that were not included in the original analyses, and the small number of studies (only three) that analysed the prevalence of the three outcomes (low muscle strength, low muscle mass and gout), whose sample sizes were not very large. However, the aim of this study was to show the prevalence of different kidney transplant-related disorders.

## Conclusion

In conclusion, this systematic review and meta-analysis shows a high prevalence regarding the presence of certain musculoskeletal disorders and their related metabolic disorders in kidney transplant recipients. Hypovitaminosis D, hyperparathyroidism and hyperuricemia were the most common metabolic disturbances. In parallel, low muscle strength, femoral osteopenia and low muscle mass were the main musculoskeletal disorders. At a clinical level, knowledge of these data will allow us to improve the prevention, diagnosis, and treatment of these complications, increase patient wellbeing, reduce the recovery time after surgery and avoid increased hospitalizations, morbidity, and mortality in kidney transplant patients, although further research is needed using experimental designs to test the effectiveness of different therapeutic prevention strategies in this specific population.

## Abbreviations

BMD, Bone mineral density; CI, Confidence intervals; FGF-23, Fibroblast growth factor 23; GODT3, Global Observatory on Donation and Transplantation; JBI, Joanna Briggs Institute; MOOSE, Meta-analyses of Observational Studies in Epidemiology; PRISMA, Preferred Reporting Items for Systematic Reviews and Meta-Analysis; PROSPERO, International Prospective Register of Systematic Reviews; PTH, Parathyroid hormone; WOS, Web of Science.
