# Supplementary material for: Prevalence of Musculoskeletal and Metabolic Disorders in Kidney Transplant Recipients: A Systematic Review and Meta-Analysis
Source: Transpl Int. 2024 Apr 24;37:12312. doi: 10.3389/ti.2024.12312 (PMC11076774; doi:10.3389/ti.2024.12312)
Supplement: Supplementary file 1 [file DataSheet1.docx]

**Supplementary material**

[**Table S1.** Search strategy. 3](#_Toc162279562)

[**Table S2.** Methodological quality of cross-sectional studies. 4](#_Toc162279563)

[**Table S3.** Methodological quality of longitudinal studies. 6](#_Toc162279564)

[**Table S4**. Comparison of the prevalences analyzed and the overall prevalences of metabolic disturbances analyzed. 7](#_Toc162279565)

[**Table S5**. Comparison of the prevalences analyzed and the overall prevalences of the musculoskeletal disorders analyzed. 7](#_Toc162279566)

[**Table S6.** Sensitivity analysis for musculoskeletal disorders 8](#_Toc162279567)

[**Table S7.** Sensitivity analysis for metabolic disorders 12](#_Toc162279568)

[**Table S8.** Metaregression analysis 14](#_Toc162279569)

[**Figure S1.** Pooled prevalence estimates for sarcopenia. 16](#_Toc162279570)

[**Figure S2.** Pooled prevalence estimates for low muscle strength. 16](#_Toc162279571)

[**Figure S3**. Pooled prevalence estimates for low muscle mass. 16](#_Toc162279572)

[**Figure S4.** Pooled prevalence estimates for osteopenia in lumbar area. 17](#_Toc162279573)

[**Figure S5.** Pooled prevalence estimates for osteopenia in femoral area. 17](#_Toc162279574)

[**Figure S6.** Pooled prevalence estimates for osteoporosis in lumbar area. 18](#_Toc162279575)

[**Figure S7.** Pooled prevalence estimates for osteoporosis in femoral area. 18](#_Toc162279575)

[**Figure S8.** Pooled prevalence estimates for fractures. 19](#_Toc162279576)

[**Figure S9.** Pooled prevalence estimates for gout. 19](#_Toc162279577)

[**Figure S10.** Pooled prevalence estimates for hypercalcemia. 20](#_Toc162279578)

[**Figure S11**. Pooled prevalence estimates for hypophosphatemia. 20](#_Toc162279579)

[**Figure S12**. Pooled prevalence estimates for hyperparathyroidism. 20](#_Toc162279579)

[**Figure S13.** Pooled prevalence estimates for hyperuricemia. 21](#_Toc162279580)

[**Figure S14**. Pooled prevalence estimates for hypovitaminosis D. 21](#_Toc162279581)

[**Figure S15.** Pooled prevalence estimates for vitamin D insufficiency. 22](#_Toc162279582)

[**Figure S16.** Pooled prevalence estimates for vitamin D deficiency. 22](#_Toc162279583)

[**Figure S17**. Comparison of the prevalence of hypercalcemia after kidney transplantation versus the overall prevalence. 23](#_Toc162279584)

[**Figure S18**. Comparison of the prevalence of hyperparathyroidism after kidney transplantation versus the overall prevalence. 23](#_Toc162279585)

[**Figure S19**. Comparison of the prevalence of hyperuricemia after kidney transplantation versus the overall prevalence. 24](#_Toc162279586)

[**Figure S20**. Comparison of the prevalence of hipovitaminosis D after kidney transplantation versus the overall prevalence. 24](#_Toc162279587)

[**Figure S21**. Comparison of the prevalence of sarcopenia after kidney transplantation versus the overall prevalence. 25](#_Toc162279588)

[**Figure S22**. Comparison of the prevalence of low muscle mass after kidney transplantation versus the overall prevalence. 25](#_Toc162279589)

[**Figure S23**. Comparison of the prevalence of osteopenia after kidney transplantation versus the overall prevalence. 26](#_Toc162279590)

[**Figure S24**. Comparison of the prevalence of osteoporosis after kidney transplantation versus the overall prevalence. 26](#_Toc162279591)

[**Figure S25**. Comparison of the prevalence of fractures after kidney transplantation versus the overall prevalence. 27](#_Toc162279592)

[**Figure S26**. Comparison of the prevalence of gout after kidney transplantation versus the overall prevalence. 27](#_Toc162279593)

# **Table S1.** Search strategy.

| “Post kidney transplant” OR “post renal transplant” OR “kidney transplant” OR “renal trasplant” |
| --- |
| **AND** |
| Musculoskeletal OR “muscular pain” OR “muscle pain” OR sarcopenia OR fibromyalgia OR myopathy OR “joint pain” OR fracture OR fragility OR “bone pain syndrome” OR “bone syndrome” OR “bone pain” OR “bone disease” OR “bone disorder” OR “lower limb pain” OR hyperparathyroidism OR hypophosphatemia OR gout OR hyperuricemia OR arthritis OR “bone loss” OR osteoporosis OR osteopenia OR osteomalacia OR “mineral disorder” OR hypercalcemia OR "vitamine D" OR “hypovitaminosis D” OR "vitamin D deficiency" |
| **AND** |
| Prevalence OR cross-sectional OR descriptive OR cohort OR prospective OR longitudinal |

# **Table S2.** Methodological quality of cross-sectional studies.

|  | Was the sample frame appropriate to address the target population? | Were study participants sampled in an appropriate way? | Was the sample size adequate? | Were the study subjects and the setting described in detail? | Was the data analysis conducted with sufficient coverage of the identified sample? | Were valid methods used for the identification of the condition? | Was the condition measured in a standard, reliable way for all participants? | Was there appropriate statistical analysis? | Was the response rate adequate, and if not, was the low response rate managed appropriately? |
| --- | --- | --- | --- | --- | --- | --- | --- | --- | --- |
| *Alagoz S. et al.^16^* |  |  |  |  | 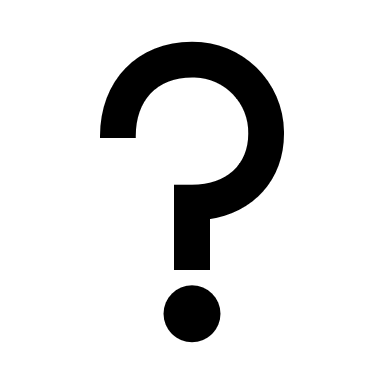 |  |  |  |  |
| *Amin T. et al.^17^* |  |  |  |  |  | 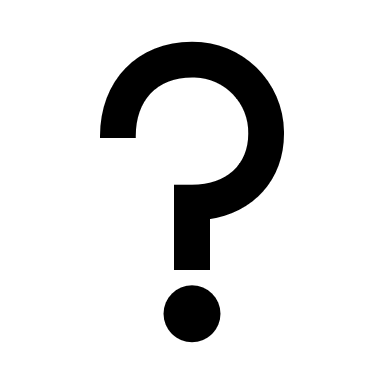 |  |  |  |
| *Berga JK. et al.^19^* |  |  |  |  | 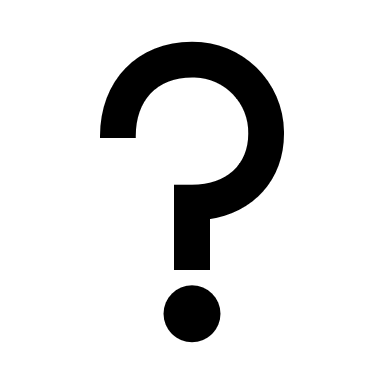 |  |  |  |  |
| *Braga Júnior JWR. et al.^20^* |  |  |  |  |  |  |  |  |  |
| *Einollahi B. et al.^23^* |  |  |  |  | 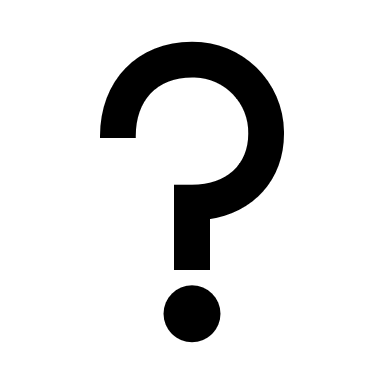 |  |  |  | 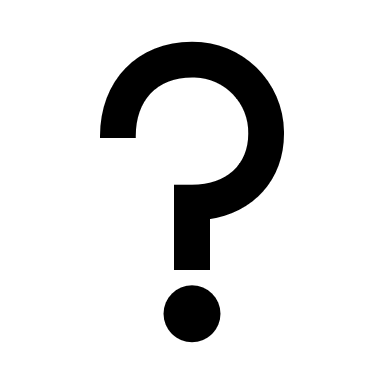 |
| *Evenepoel P. et al.^24^* |  |  |  |  | 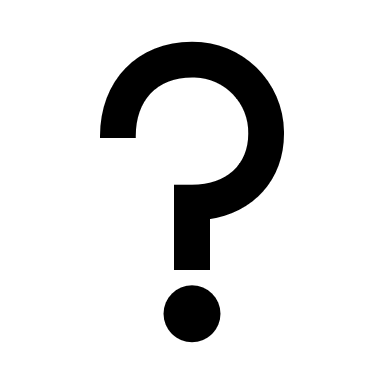 |  |  |  | 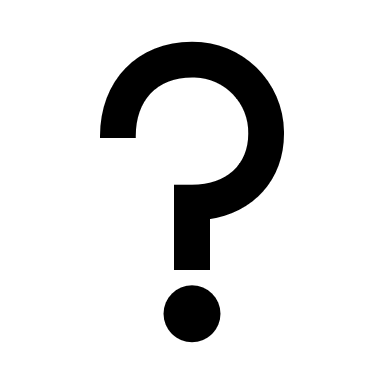 |
| *Fernández Castillo RF. et al.^25^* |  |  |  |  |  |  |  |  |  |
| *Gregorini M. et al.^26^* |  |  |  |  |  | 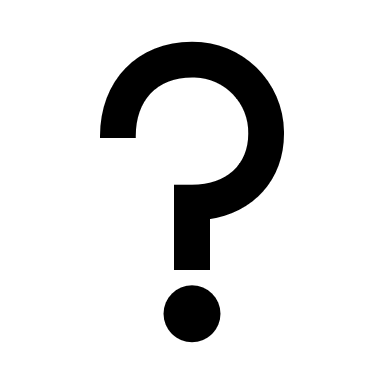 |  |  | 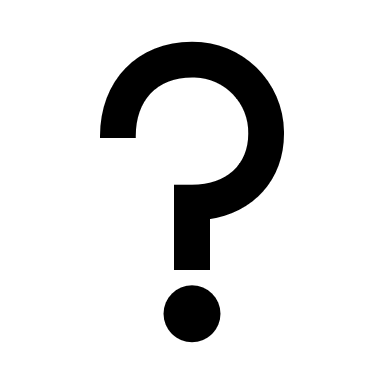 |
| *Jerman A. et al.^28^* |  |  |  |  |  |  |  |  |  |
| *Jørgensen HS. et al.^29^* |  |  |  |  |  |  |  |  |  |
| *Khosravi M. et al.^30^* |  |  |  |  |  |  |  |  |  |
| *Kim KM. et al.^31^* |  |  |  |  | 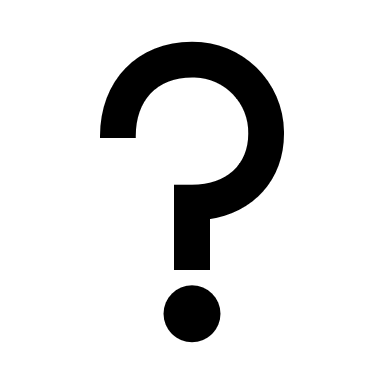 |  |  |  |  |
| *Kosoku A. et al.^32^* |  |  |  |  |  |  |  |  |  |
| *Limirio LS. et al.^33^* |  |  |  |  |  |  |  |  |  |
| *López Ruiz ML. et al.^34^* |  |  |  |  |  |  |  |  |  |
| *Malheiro J. et al.^35^* |  |  |  |  | 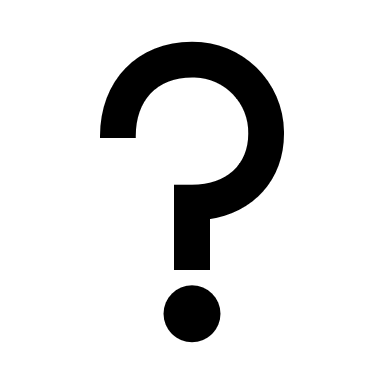 |  |  |  |  |
| *Marcén R. et al.^36^* |  |  |  |  |  |  |  |  |  |
| *Menna Barreto APM. et al.^37^* |  |  |  |  |  |  |  |  | 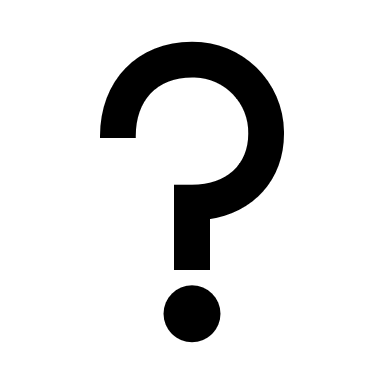 |
| *Ozkayar N. et al.^39^* | 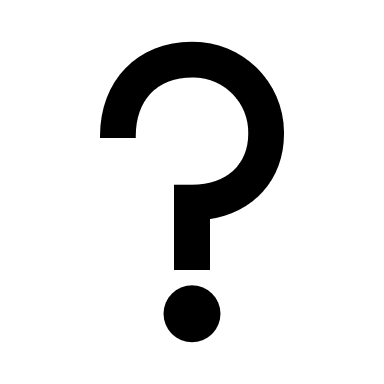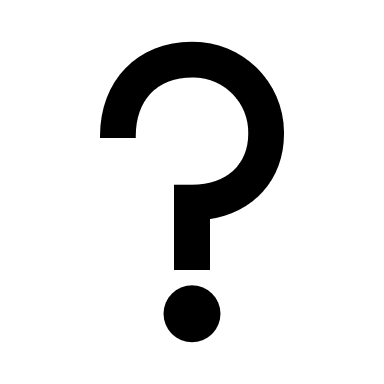 |  |  |  |  |  |  |  |  |
| *Park WY. et al.^40^* |  |  |  |  |  |  |  |  |  |
| *Patel S. et al.^41^* |  |  |  |  |  |  |  |  |  |
| *Savaj S. et al.^42^* |  |  |  |  |  |  |  |  |  |
| *Stamp L. et al.^46^* |  |  |  |  |  |  |  |  |  |
| *Torres A. et al.^47^* |  |  |  |  |  |  |  |  |  |
| *Velioglu A. et al.^48^* |  |  |  |  |  |  |  |  |  |
| *Vilarta CF. et al.^49^* |  |  |  |  |  |  |  |  |  |
| *Wang C. et al.^50^* |  |  |  |  |  |  |  |  |  |


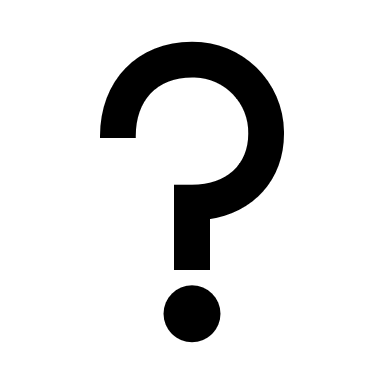


YES= NO= UNCLEAR=

# **Table S3.** Methodological quality of longitudinal studies.

|  | Were the two groups similar and recruited from the same population? | Were the exposures measured similarly to assign people to both exposed and unexposed groups? | Was the exposure measured in a valid and reliable way? | Was the exposure measured in a valid and reliable way? | Were strategies to deal with confounding factors stated? | Were the groups/participants free of the outcome at the start of the study (or at the moment of exposure)? | Were the outcomes measured in a valid and reliable way? | Was the follow up time reported and sufficient to be long enough for outcomes to occur? | Was follow up complete, and if not, were the reasons to loss to follow up described and explored? | Were strategies to address incomplete follow up utilized? | Was appropriate statistical analysis used? |
| --- | --- | --- | --- | --- | --- | --- | --- | --- | --- | --- | --- |
| *Batteux B. et al.^18^* |  | 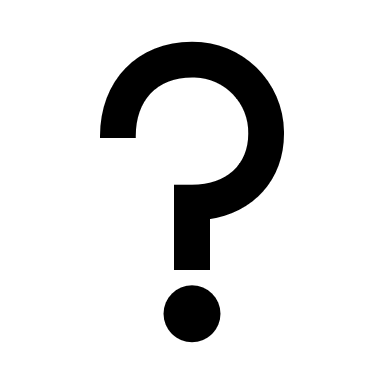 |  |  |  |  |  |  |  | 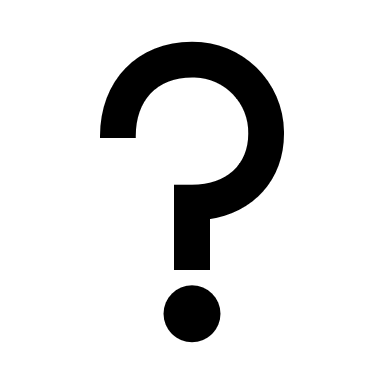 |  |
| *Chan W. et al.^21^* | 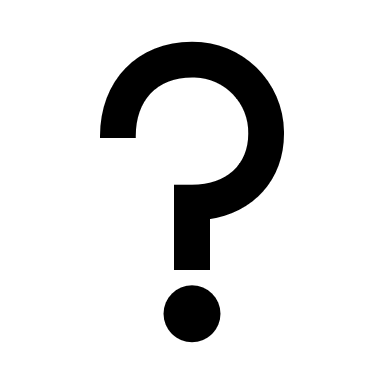 | 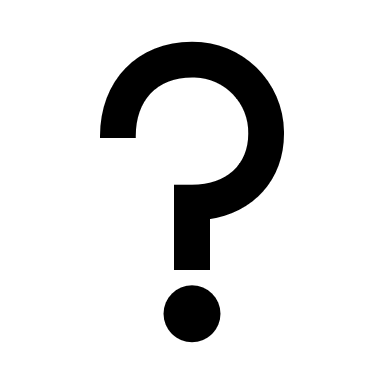 |  |  |  |  |  |  |  |  |  |
| *Conley E. et al.^22^* |  |  |  | 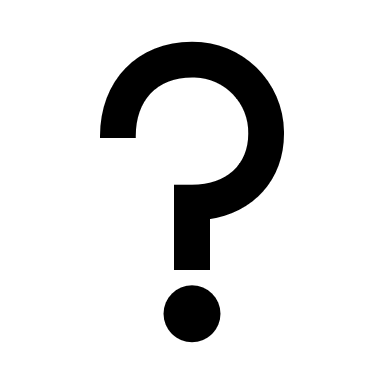 |  |  |  |  |  | 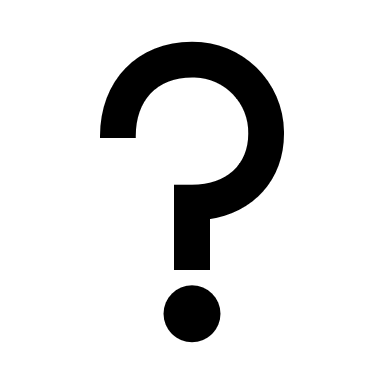 |  |
| *Hamidian Jahromi A. et al.^27^* |  |  |  |  |  |  |  | 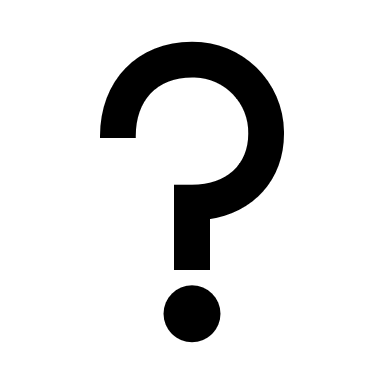 |  |  |  |
| *Muirhead N. et al^38^* |  |  | 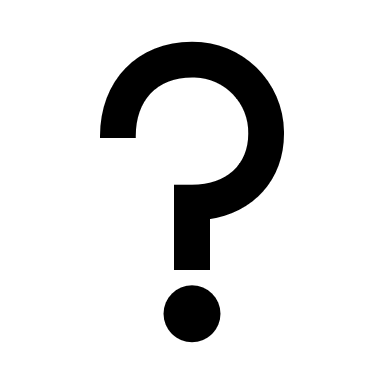 |  |  |  |  |  |  | 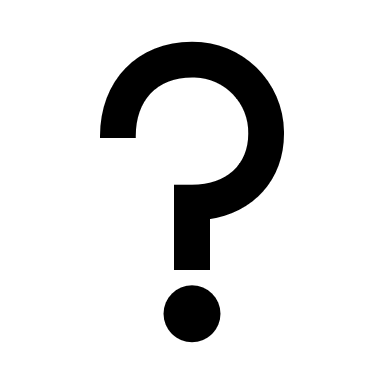 |  |
| *Schreiber PW. et al.^43^* |  |  |  |  |  |  |  |  |  |  |  |
| *Segaud N. et al.^44^* |  |  |  |  |  |  |  |  |  |  |  |
| *Simbolon FR. et al.^45^* |  |  |  |  |  |  |  |  |  |  |  |
| *Weng SC. et al.^51^* |  |  |  |  |  |  |  |  |  |  |  |
| *Wolf M. et al.^52^* |  |  |  |  |  |  |  |  |  |  |  |
| *Zhang K. et al.^53^* |  |  |  |  |  |  |  |  |  |  |  |


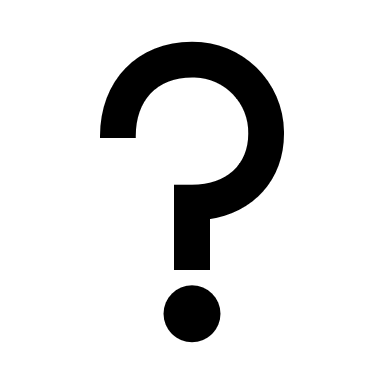


YES= NO= UNCLEAR=

# **Table S4**. Comparison of the prevalences analyzed and the overall prevalences of metabolic disturbances analyzed.

| **Variables analyzed** | **Total prevalence analyzed (%)** | **CI 95% (%)** | **Overall prevalence (%)** | **CI 95%**  **(%)** |
| --- | --- | --- | --- | --- |
| Hypercalcemia | 14.9 | 10.4-21.0 | 0.8 | 0.6-0.9 |
| Hypophosphatemia | 13.8 | 3.1-44.5 | - | - |
| Hiperparathyroidism | 58.0 | 40.5-73.7 | 0.8 | 0.7-1.0 |
| Hyperuricemia | 31.2 | 24.0-39.5 | 13.3 | 11.9-14.6 |
| Hypovitaminosis D | 81.8 | 67.2-90.8 | 15.7 | 13.7-17.8 |

#

# **Table S5**. Comparison of the prevalences analyzed and the overall prevalences of the musculoskeletal disorders analyzed.

| **Variables analyzed** | | **Total prevalence analyzed (%)** | **CI 95%**  **(%)** | **Overall prevalence (%)** | **CI 95%**  **(%)** |
| --- | --- | --- | --- | --- | --- |
| Sarcopenia | | 23.6 | 13.2-38.5 | 15.5 | 11.8-19.1 |
| Low muscle strenght | | 64.5 | 43.1-81.3 | - | - |
| Low muscle mass | | 39.5 | 20.3-62.6 | 27.0 | 23.0-31.0 |
| Osteopenia | Lumbar area | 30.7 | 23.3-39.3 | 40.4 | 36.9-43.8 |
|  | Femoral area | 42.6 | 36.5-48.8 |  |  |
| Osteoporosis | Lumbar area | 13.8 | 10.4-17.9 | 18.3 | 16.2-20.7 |
|  | Femoral area | 19.2 | 13.4-26.7 |  |  |
| Fractures | | 14.2 | 9.1-21.5 | 1.1 | 1.0-1.2 |
| Gout | | 15.4 | 8.3-26.9 | 1.1 | 0.7-1.5 |

# **Table S6.** Sensitivity analysis for musculoskeletal disorders

| Sarcopenia | Study removed | Proportion | Lower limit | Upper Limit |
| --- | --- | --- | --- | --- |
|  | Chan W. [21] | 0.242 | 0.209 | 0.279 |
|  | Kosoku A. [32] | 0.287 | 0.251 | 0.327 |
|  | Limirio LS. [33] | 0.191 | 0.163 | 0.223 |
|  | Menna Barreto ANP. [37] | 0.274 | 0.238 | 0.314 |
|  | Ozkayar N. [39] | 0.264 | 0.229 | 0.303 |
| Low muscle strength | Study removed | Proportion | Lower limit | Upper Limit |
|  | Chan W. [21] | 0.578 | 0.518 | 0.635 |
|  | Limirio LS. [33] | 0.532 | 0.475 | 0.587 |
|  | Menna Barreto ANP. [37] | 0.714 | 0.654 | 0.767 |
| Low muscle mass | Study removed | Proportion | Lower limit | Upper Limit |
|  | Chan W. [21] | 0.403 | 0.345 | 0.463 |
|  | Limirio LS. [33] | 0.291 | 0.243 | 0.344 |
|  | Menna Barreto ANP. [37] | 0.487 | 0.424 | 0.551 |
| Osteopenia in lumbar area | Study removed | Proportion | Lower limit | Upper Limit |
|  | Batteux B. [18] | 0.317 | 0.299 | 0.337 |
|  | Braga Jr JWR. [20] | 0.320 | 0.302 | 0.339 |
|  | Evenepoel P. [24] | 0.347 | 0.329 | 0.367 |
|  | Fernández Castillo R. [25] | 0.320 | 0.302 | 0.339 |
|  | Gregorini M. [26] | 0.310 | 0.291 | 0.329 |
|  | Jorgensen HS. [29] | 0.307 | 0.287 | 0.328 |
|  | Khosravi M. [30] | 0.310 | 0.292 | 0.328 |
|  | López Ruiz ML. [34] | 0.337 | 0.318 | 0.356 |
|  | Patel S. [41] | 0.321 | 0.303 | 0.340 |
|  | Savaj S. [42] | 0.311 | 0.294 | 0.330 |
|  | Velioglu A. [48] | 0.323 | 0.305 | 0.342 |
| Osteopenia in femoral area | Study removed | Proportion | Lower limit | Upper Limit |
|  | Batteux B. [18] | 0.415 | 0.351 | 0.482 |
|  | Braga Jr JWR. [20] | 0.435 | 0.372 | 0.499 |
|  | Evenepoel P. [24] | 0.414 | 0.351 | 0.479 |
|  | Fernández Castillo R. [25] | 0.420 | 0.356 | 0.486 |
|  | Gregorini M. [26] | 0.418 | 0.353 | 0.487 |
|  | Jorgensen HS. [29] | 0.416 | 0.350 | 0.486 |
|  | López Ruiz ML. [34] | 0.453 | 0.410 | 0.497 |
|  | Park WY. [40] | 0.428 | 0.363 | 0.495 |
|  | Patel S. [41] | 0.427 | 0.363 | 0.494 |
|  | Savaj S. [42] | 0.431 | 0.368 | 0.496 |
|  | Segaud N. [44] | 0.425 | 0.360 | 0.493 |
|  | Velioglu A. [48] | 0.427 | 0.362 | 0.494 |
| Osteoporosis in lumbar area | Study removed | Proportion | Lower limit | Upper Limit |
|  | Batteux B. [18] | 0.149 | 0.116 | 0.190 |
|  | Braga Jr JWR. [20] | 0.140 | 0.104 | 0.185 |
|  | Evenepoel P. [24] | 0.129 | 0.097 | 0.169 |
|  | Fernández Castillo R. [25] | 0.126 | 0.096 | 0.165 |
|  | Gregorini M. [26] | 0.137 | 0.101 | 0.183 |
|  | Jorgensen HS. [29] | 0.133 | 0.095 | 0.183 |
|  | Khosravi M. [30] | 0.133 | 0.098 | 0.177 |
|  | López Ruiz ML. [34] | 0.139 | 0.103 | 0.185 |
|  | Patel S. [41] | 0.144 | 0.109 | 0.188 |
|  | Savaj S. [42] | 0.139 | 0.104 | 0.183 |
|  | Velioglu A. [48] | 0.145 | 0.110 | 0.189 |
| Osteoporosis in femoral area. | Study removed | Proportion | Lower limit | Upper Limit |
|  | Batteux B. [18] | 0.203 | 0.142 | 0.282 |
|  | Braga Jr JWR. [20] | 0.201 | 0.139 | 0.281 |
|  | Evenepoel P. [24] | 0.188 | 0.124 | 0.276 |
|  | Fernández Castillo R. [25] | 0.196 | 0.134 | 0.276 |
|  | Gregorini M. [26] | 0.190 | 0.127 | 0.273 |
|  | Jorgensen HS. [29] | 0.185 | 0.120 | 0.275 |
|  | López Ruiz ML. [34] | 0.209 | 0.148 | 0.286 |
|  | Park WY. [40] | 0.174 | 0.121 | 0.242 |
|  | Patel S. [41] | 0.202 | 0.140 | 0.282 |
|  | Savaj S. [42] | 0.175 | 0.120 | 0.248 |
|  | Segaud N. [44] | 0.177 | 0.121 | 0.250 |
|  | Velioglu A. [48] | 0.205 | 0.143 | 0.285 |
| Fractures | Study removed | Proportion | Lower limit | Upper Limit |
|  | Braga Jr JWR. [20] | 0.134 | 0.083 | 0.210 |
|  | Conley E. [22] | 0.143 | 0.086 | 0.228 |
|  | Evenepoel P. [24] | 0.152 | 0.096 | 0.232 |
|  | Gregorini M. [26] | 0.144 | 0.089 | 0.226 |
|  | Jerman A. [28] | 0.144 | 0.087 | 0.228 |
|  | Patel S. [41] | 0.140 | 0.087 | 0.219 |
|  | Segaud N. [44] | 0.146 | 0.091 | 0.227 |
|  | Torres A. [47] | 0.141 | 0.084 | 0.229 |
|  | Velioglu A. [48] | 0.120 | 0.095 | 0.150 |
|  | Vilarta CF. [49] | 0.147 | 0.092 | 0.226 |
|  | Wang C. [50] | 0.160 | 0.103 | 0.240 |
| Gout | Study removed | Proportion | Lower limit | Upper Limit |
|  | Simbolon FR. [45] | 0.199 | 0.151 | 0.257 |
|  | Stamp L. [46] | 0.126 | 0.061 | 0.240 |
|  | Weng SC. [51] | 0.145 | 0.052 | 0.342 |

# **Table S7.** Sensitivity analysis for metabolic disorders

| Hypercalcemia | Study removed | Proportion | Lower limit | Upper Limit |
| --- | --- | --- | --- | --- |
|  | Alagoz S. [16] | 0.144 | 0.095 | 0.212 |
|  | Amin T. [17] | 0.148 | 0.093 | 0.229 |
|  | Jahromi AH. [27] | 0.145 | 0.097 | 0.213 |
|  | Muirhead N. [38] | 0.146 | 0.089 | 0.230 |
|  | Torres A. [47] | 0.171 | 0.129 | 0.225 |
|  | Wang C. [50] | 0.161 | 0.109 | 0.230 |
|  | Wolf M. [52] | 0.130 | 0.094 | 0.177 |
| Hypophosphatemia | Study removed | Proportion | Lower limit | Upper Limit |
|  | Alagoz S. [16] | 0.127 | 0.015 | 0.581 |
|  | Torres A. [47] | 0.179 | 0.037 | 0.554 |
|  | Wang C. [50] | 0.203 | 0.039 | 0.612 |
|  | Wolf M. [52] | 0.077 | 0.032 | 0.177 |
| Hyperparathyroidism | Study removed | Proportion | Lower limit | Upper Limit |
|  | Alagoz S. [16] | 0.600 | 0.399 | 0.772 |
|  | Jahromi AH. [27] | 0.671 | 0.507 | 0.802 |
|  | Muirhead N. [38] | 0.596 | 0.380 | 0.780 |
|  | Savaj S. [42] | 0.546 | 0.356 | 0.723 |
|  | Torres A. [47] | 0.543 | 0.357 | 0.717 |
|  | Velioglu A. [48] | 0.588 | 0.386 | 0.764 |
|  | Wolf M. [52] | 0.507 | 0.339 | 0.673 |
| Hyperuricemia | Study removed | Proportion | Lower limit | Upper Limit |
|  | Einollahi E. [23] | 0.309 | 0.199 | 0.447 |
|  | Kim KM. [31] | 0.359 | 0.287 | 0.438 |
|  | Malheiro J. [35] | 0.288 | 0.211 | 0.381 |
|  | Weng SC. [51] | 0.283 | 0.212 | 0.366 |
|  | Zhang K. [53] | 0.323 | 0.235 | 0.426 |
| Hypovitaminosis D | Study removed | Proportion | Lower limit | Upper Limit |
|  | Berga JK. [19] | 0.783 | 0.616 | 0.891 |
|  | Evenepoel P. [24] | 0.841 | 0.784 | 0.886 |
|  | Marcén R. [36] | 0.813 | 0.644 | 0.913 |
|  | Savaj S. [42] | 0.789 | 0.623 | 0.895 |
|  | Torres A. [47] | 0.817 | 0.642 | 0.918 |
|  | Velioglu A. [48] | 0.834 | 0.672 | 0.924 |
|  | Vilarta CF. [49] | 0.822 | 0.658 | 0.917 |
|  | Wang C. [50] | 0.823 | 0.655 | 0.919 |
| Vitamin D insufficiency | Study removed | Proportion | Lower limit | Upper Limit |
|  | Berga JK. [19] | 0.783 | 0.616 | 0.891 |
|  | Evenepoel P. [24] | 0.841 | 0.784 | 0.886 |
|  | Marcén R. [36] | 0.813 | 0.644 | 0.913 |
|  | Savaj S. [42] | 0.789 | 0.623 | 0.895 |
|  | Torres A. [47] | 0.817 | 0.642 | 0.918 |
|  | Velioglu A. [48] | 0.834 | 0.673 | 0.925 |
|  | Vilarta CF. [49] | 0.822 | 0.658 | 0.917 |
|  | Wang C. [50] | 0.823 | 0.656 | 0.919 |
| Vitamin D deficiency | Study removed | Proportion | Lower limit | Upper Limit |
|  | Berga JK. [19] | 0.306 | 0.208 | 0.427 |
|  | Evenepoel P. [24] | 0.400 | 0.323 | 0.483 |
|  | Marcén R. [36] | 0.321 | 0.205 | 0.464 |
|  | Savaj S. [42] | 0.314 | 0.212 | 0.439 |
|  | Schreiber W. [43] | 0.293 | 0.205 | 0.401 |
|  | Torres A. [47] | 0.328 | 0.207 | 0.476 |
|  | Velioglu A. [48] | 0.349 | 0.242 | 0.473 |
|  | Vilarta CF. [49] | 0.318 | 0.213 | 0.445 |
|  | Wang C. [50] | 0.328 | 0.219 | 0.460 |

# **Table S8.** Metaregression analysis

| **Osteopenia (lumbar area)** | β 95% CI) | p |
| --- | --- | --- |
| % females | 0.007 (-0.004 to 0.020) | 0.202 |
| Participants mean age | -0.003 (-0.011 to 0.003) | 0.307 |
| Time since transplant | 0.005 (0.003 to 0.007) | <0.001 |
| Time on haemodialysis prior to transplant | -0.009 (-0.018 to -0.001) | 0.043 |
| **Osteopenia (femoral area)** | β (95% CI) | p |
| % females | -0.030 (-0.040 to -0.010) | <0.001 |
| Participants mean age | 0.085 (0.066 to 0.104) | <0.001 |
| Time since transplant | -0.006 (-0.008 to -0.003) | <0.001 |
| Time on haemodialysis prior to transplant | -0.015 (-0.02 to -0.007) | <0.001 |
| **Osteoporosis (lumbar area)** | β (95% CI) | p |
| % females | -0.009 (-0.026 to 0.007) | 0.266 |
| Participants mean age | 0.057 (0.031 to 0.089) | <0.001 |
| Time since transplant | -0.006 (-0.009 to -0.003) | <0.001 |
| Time on haemodialysis prior to transplant | -0.003 (-0.007 to 0.008) | 0.119 |
| **Fractures** | β (95% CI) | p |
| % females | 0.084 (0.067 to 0.102) | <0.001 |
| Participants mean age | 0.056 (0.035 to 0.078) | <0.001 |
| Time since transplant | 0.007 (0.005 to 0.105) | <0.001 |
| Time on haemodialysis prior to transplant | -0.023 (-0.031 to -0.016) | <0.001 |


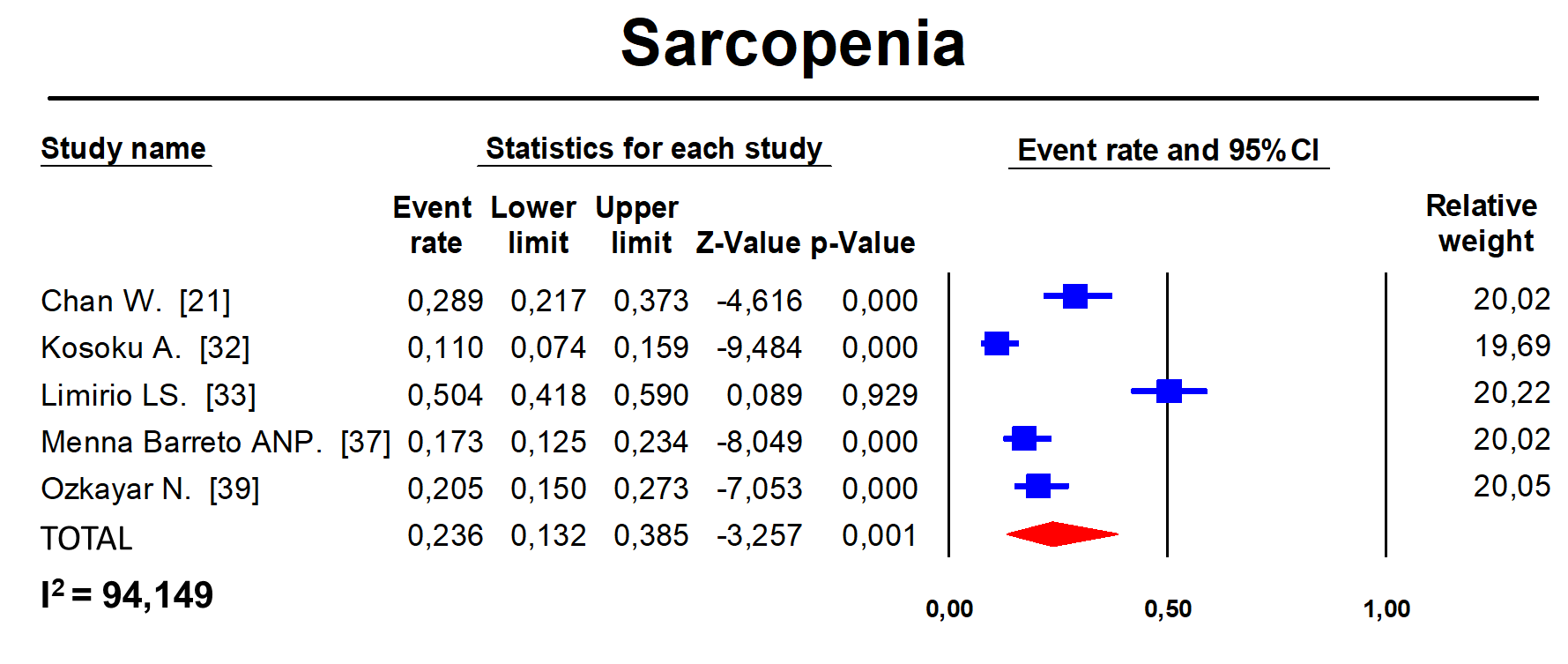

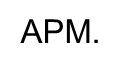


# **Figure S1.** Pooled prevalence estimates for sarcopenia.


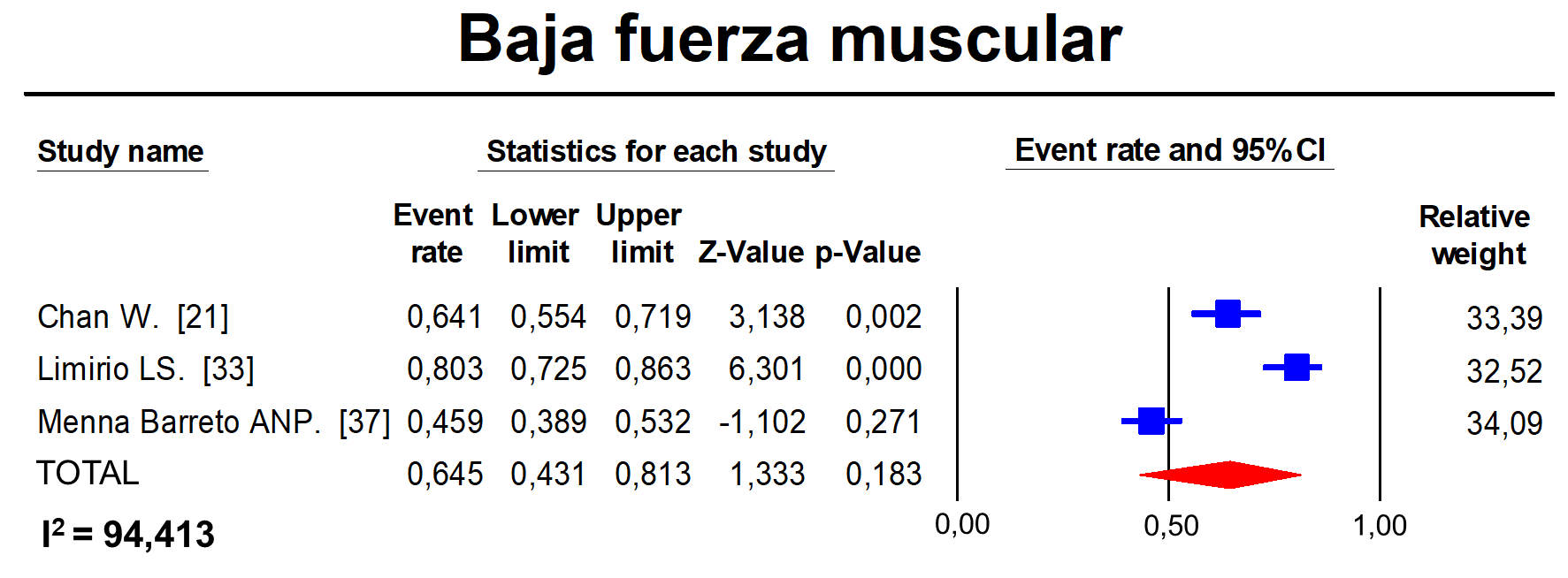

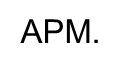


# **Figure S2.** Pooled prevalence estimates for low muscle strength.


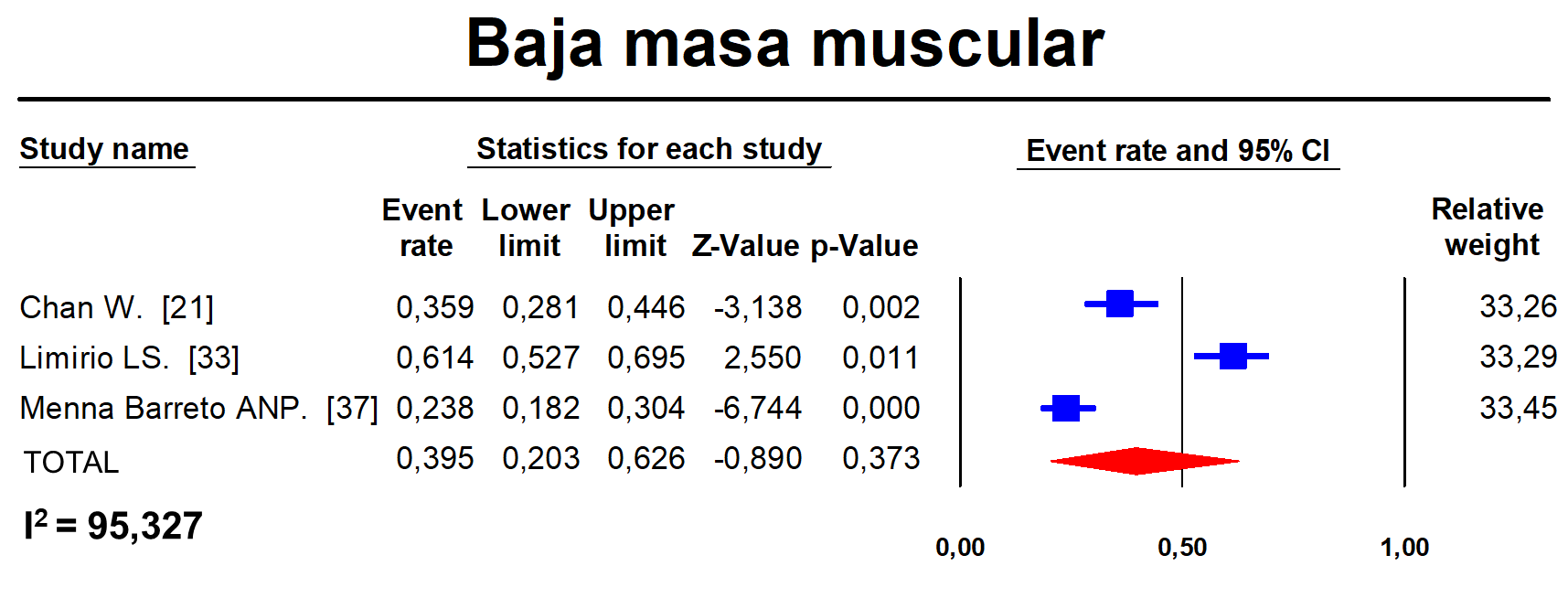

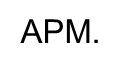


# **Figure S3**. Pooled prevalence estimates for low muscle mass.

# **Figure S4.** Pooled prevalence estimates for osteopenia in lumbar area.
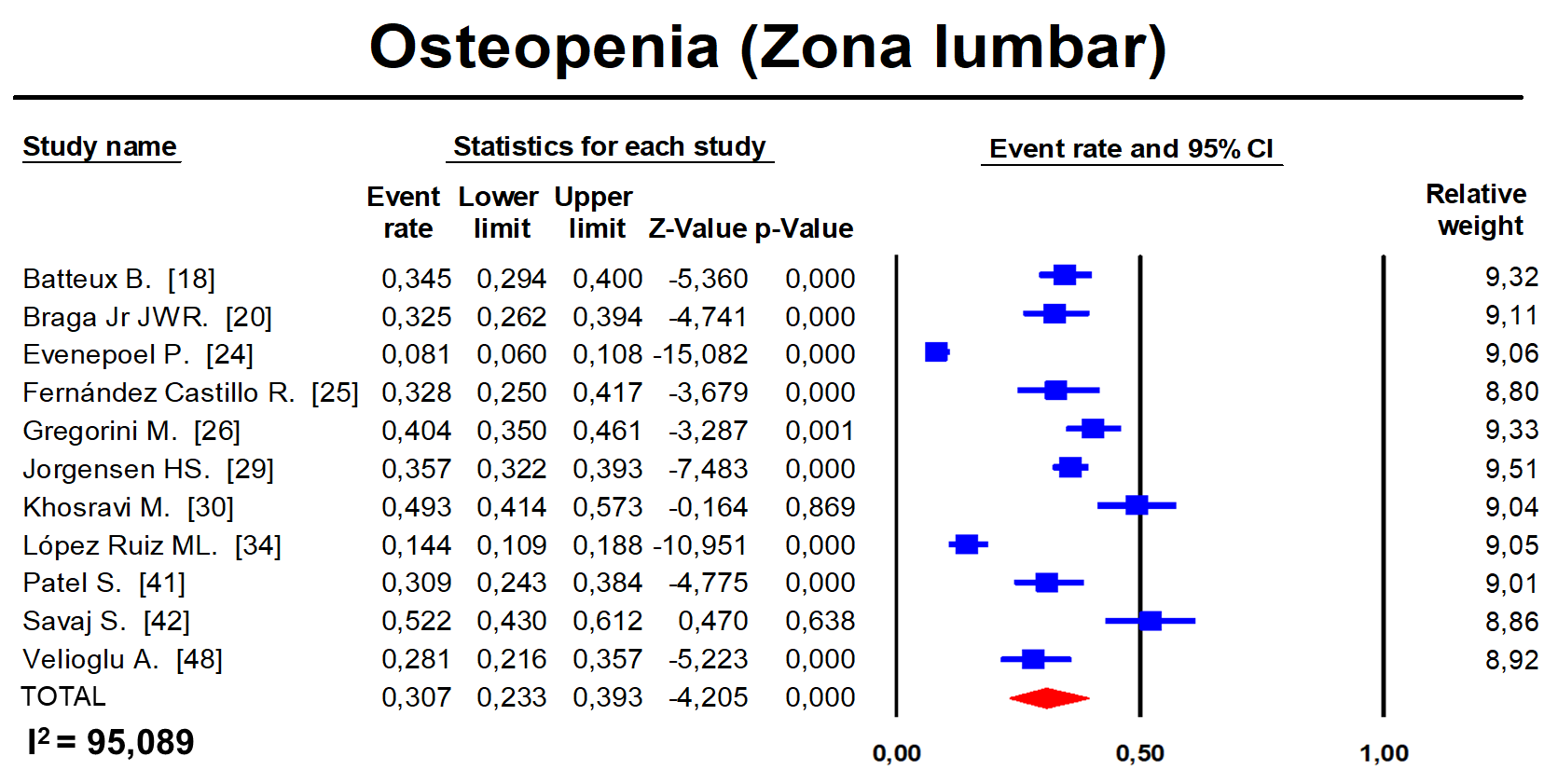


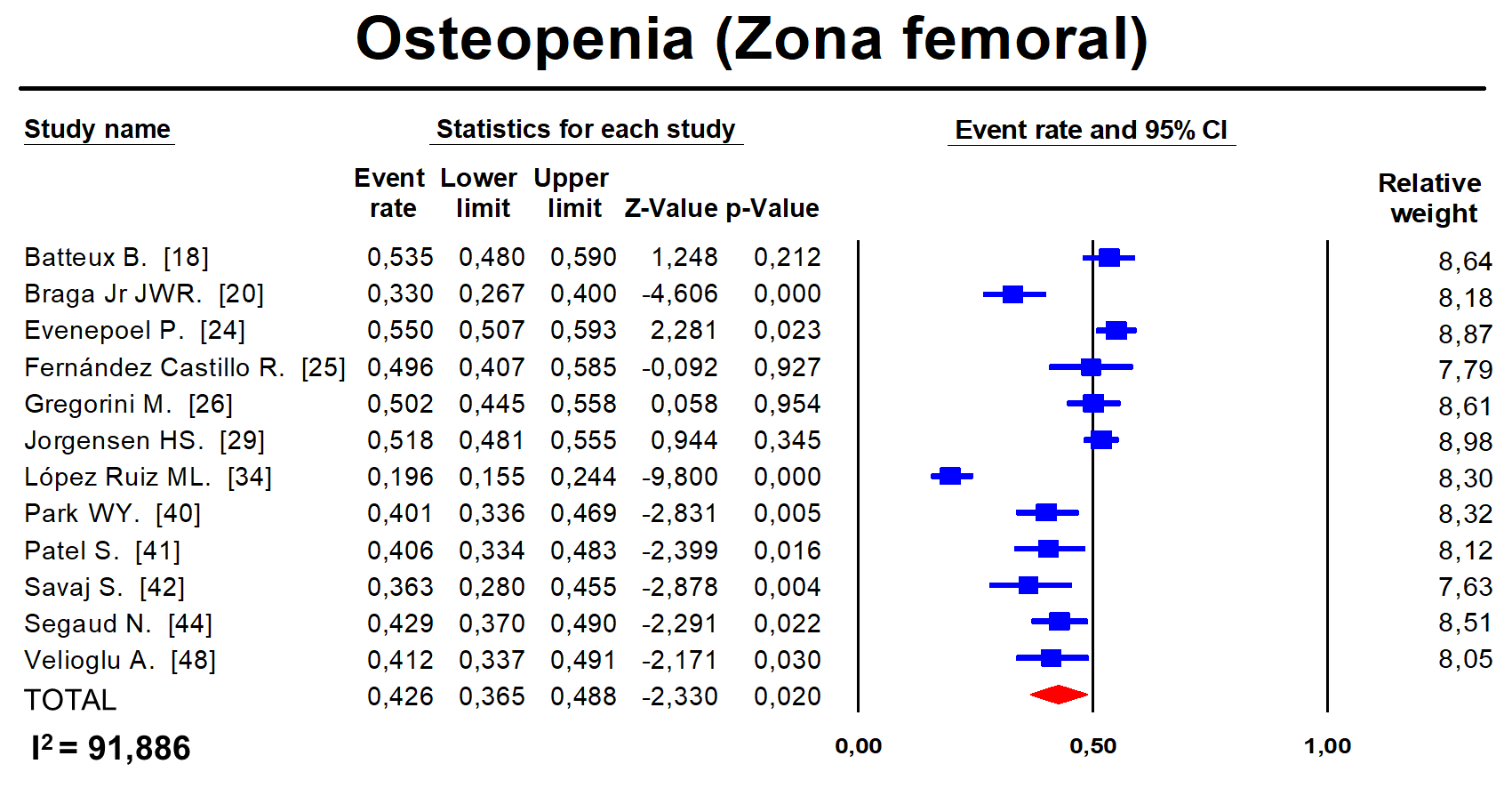


# **Figure S5.** Pooled prevalence estimates for osteopenia in femoral area.


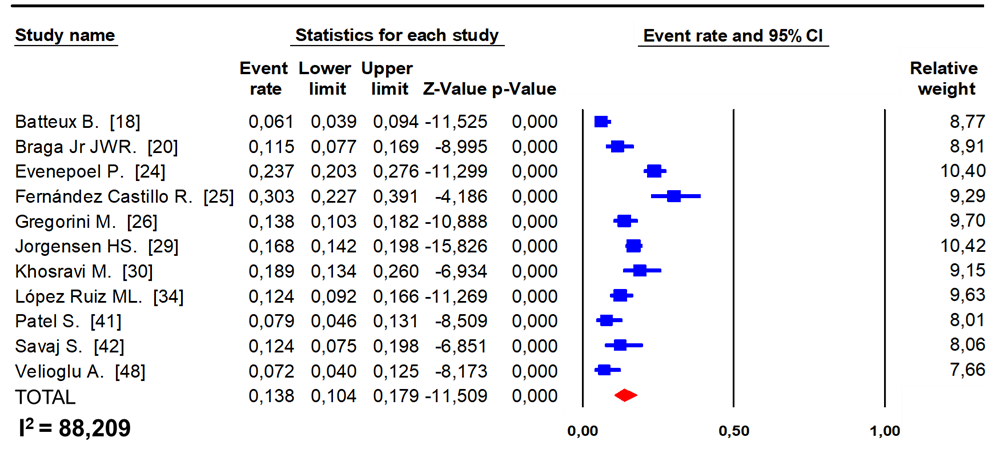


# **Figure S6.** Pooled prevalence estimates for osteoporosis in lumbar area.

**
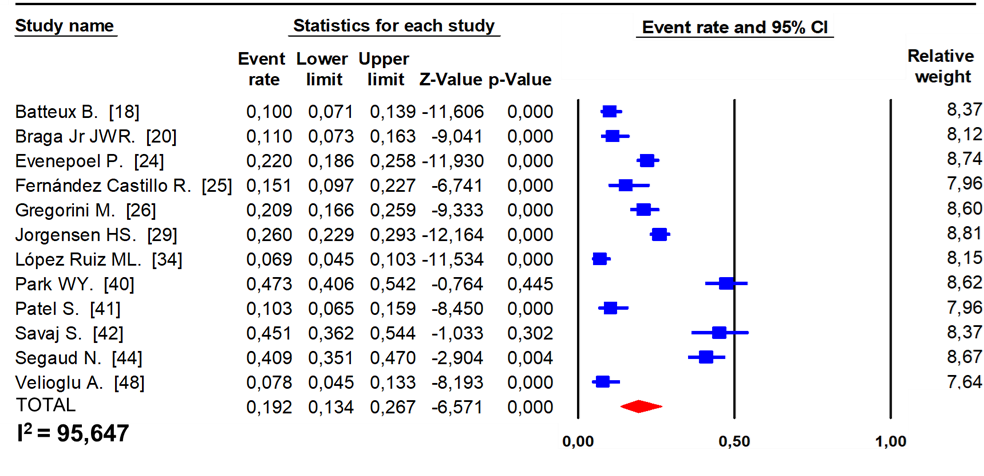
Figure S7.** Pooled prevalence estimates for osteoporosis in femoral area.

**
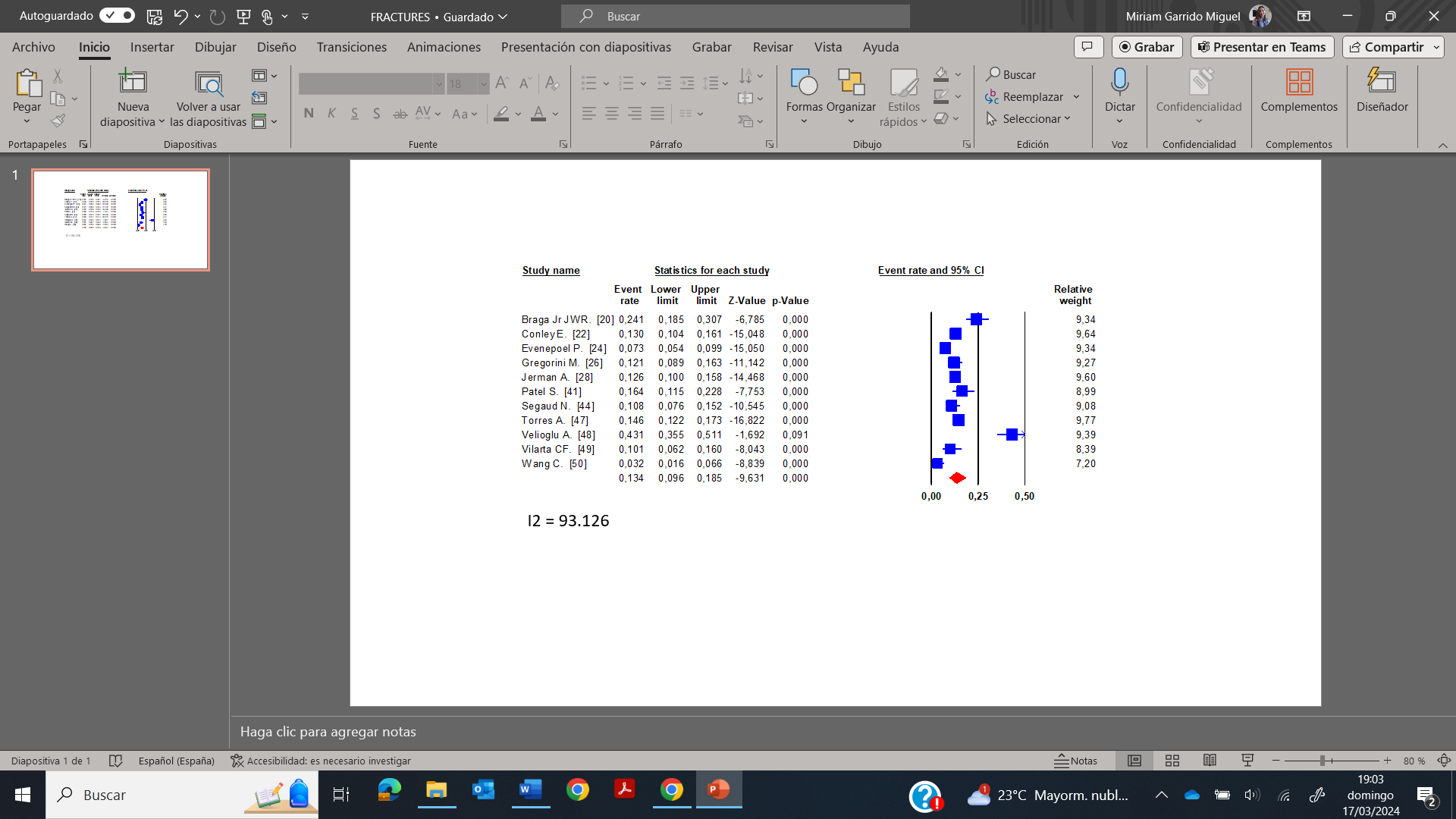
**

# **Figure S8.** Pooled prevalence estimates for fractures.


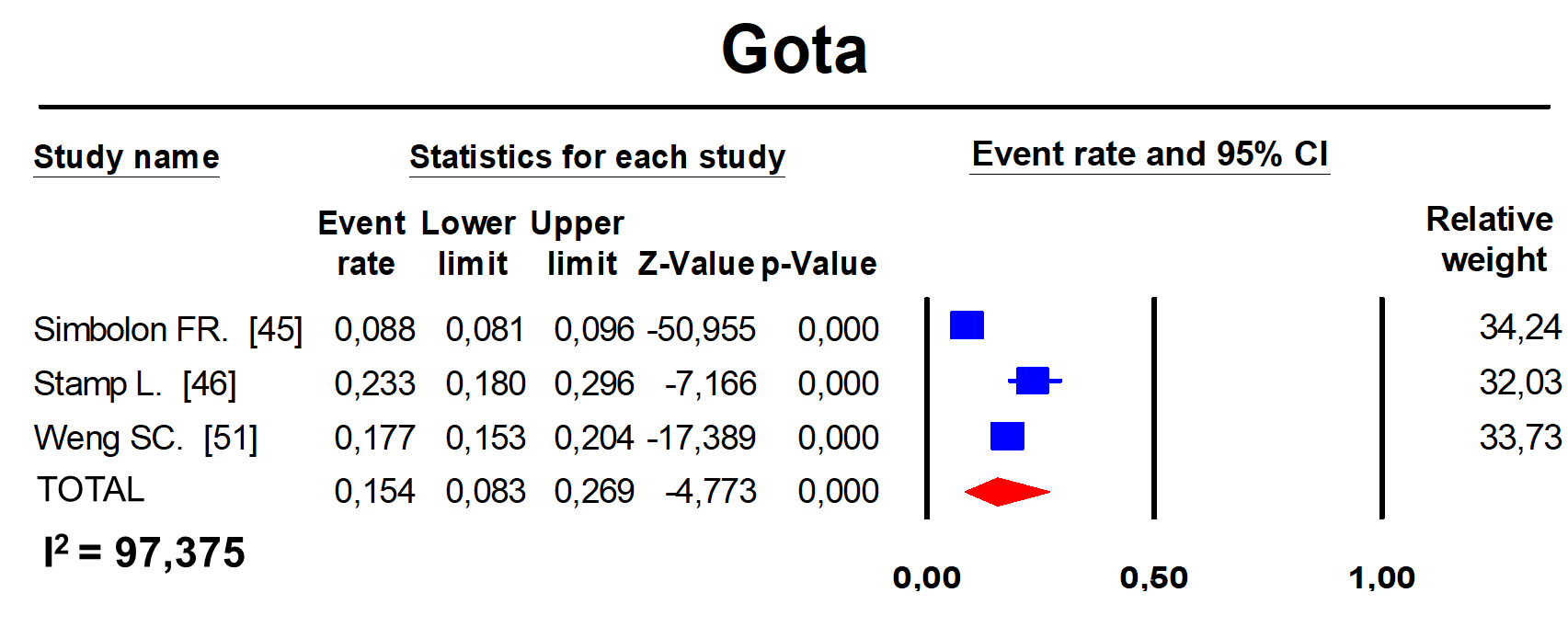


# **Figure S9.** Pooled prevalence estimates for gout.


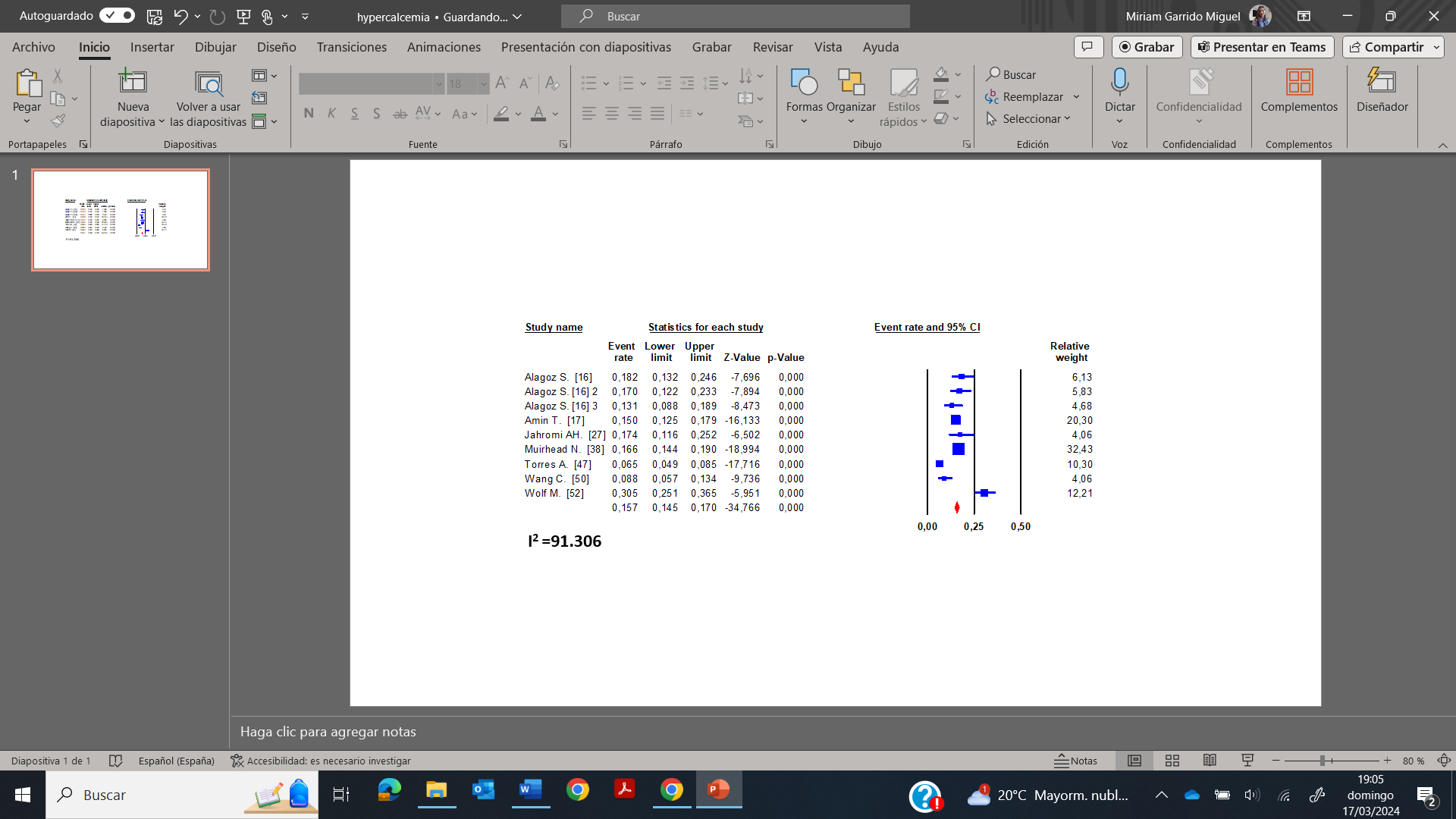


# **Figure S10.** Pooled prevalence estimates for hypercalcemia.


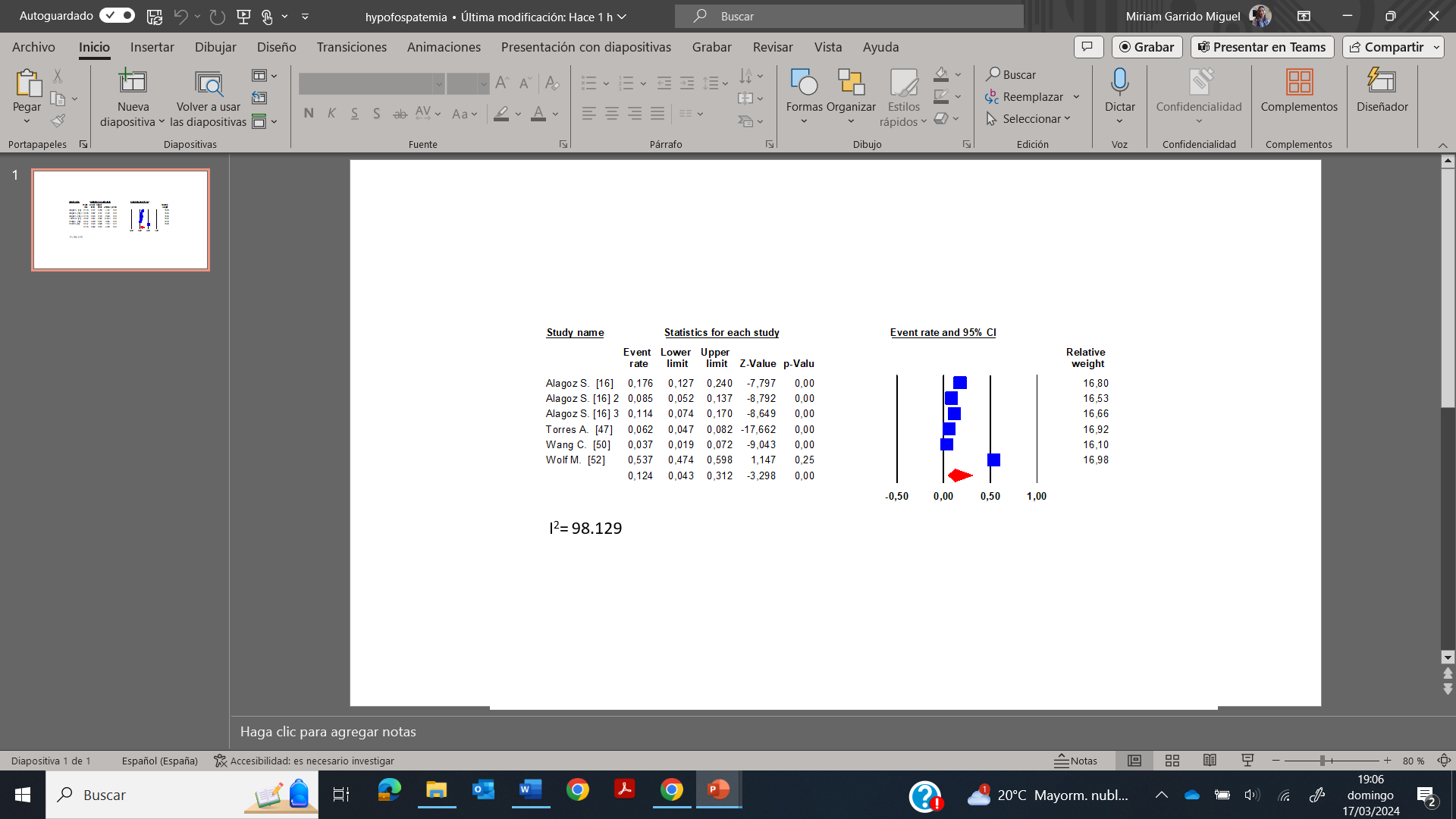


# **Figure S11**. Pooled prevalence estimates for hypophosphatemia.


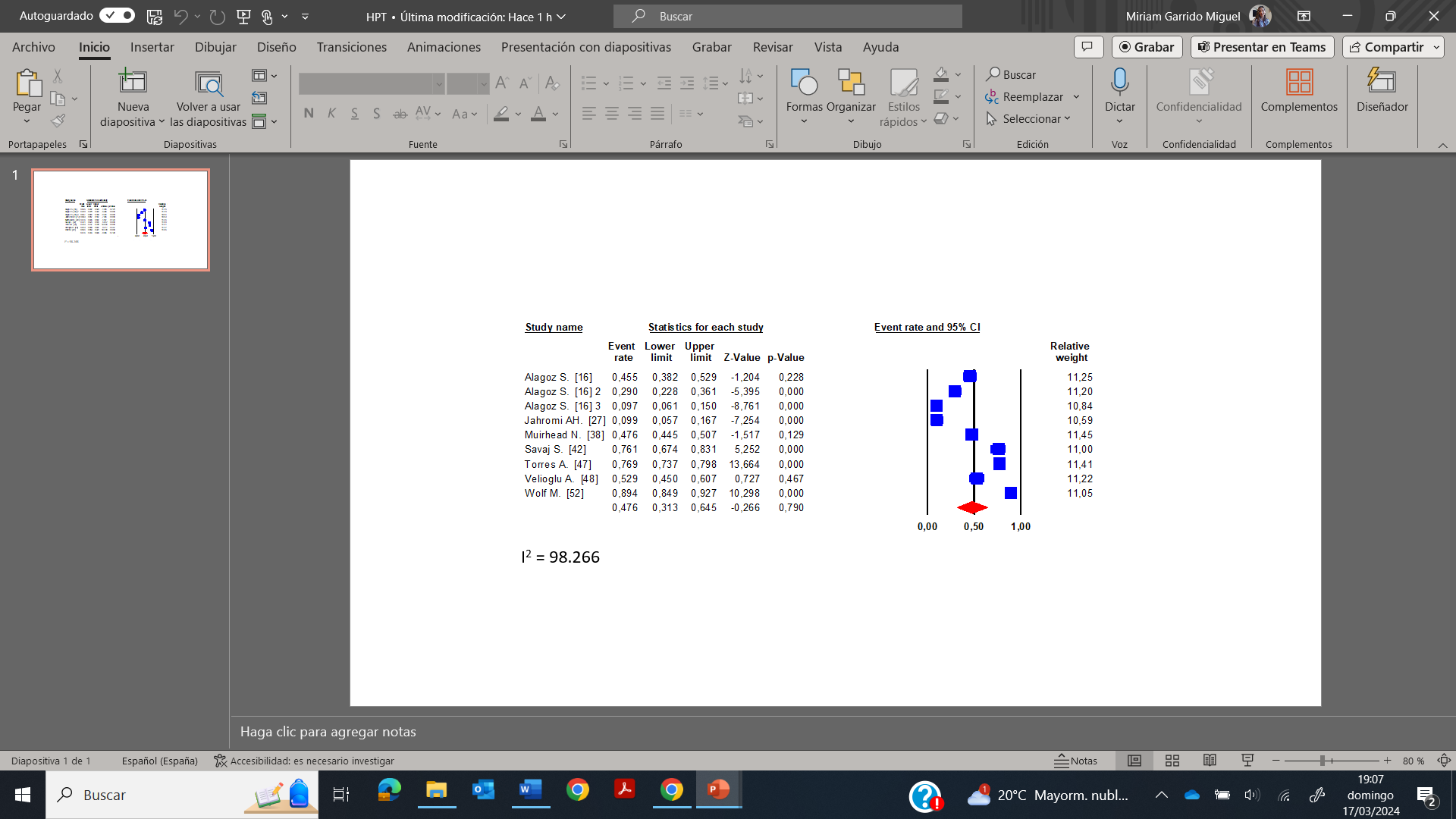
**Figure S12.** Pooled prevalence estimates for hyperparathyroidism.


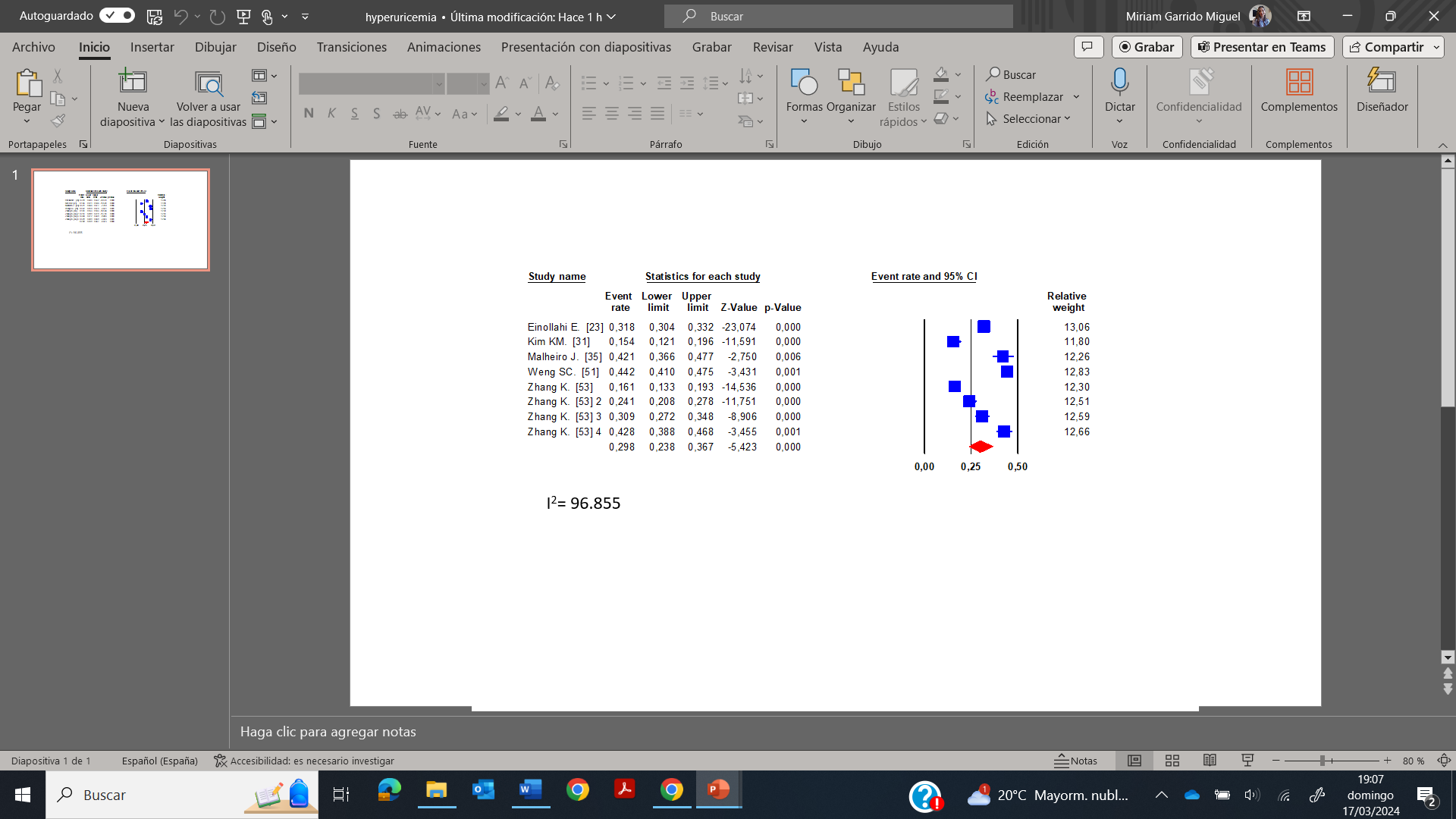


# **Figure S13.** Pooled prevalence estimates for hyperuricemia.

# **Figure S14**. Pooled prevalence estimates for hypovitaminosis D.
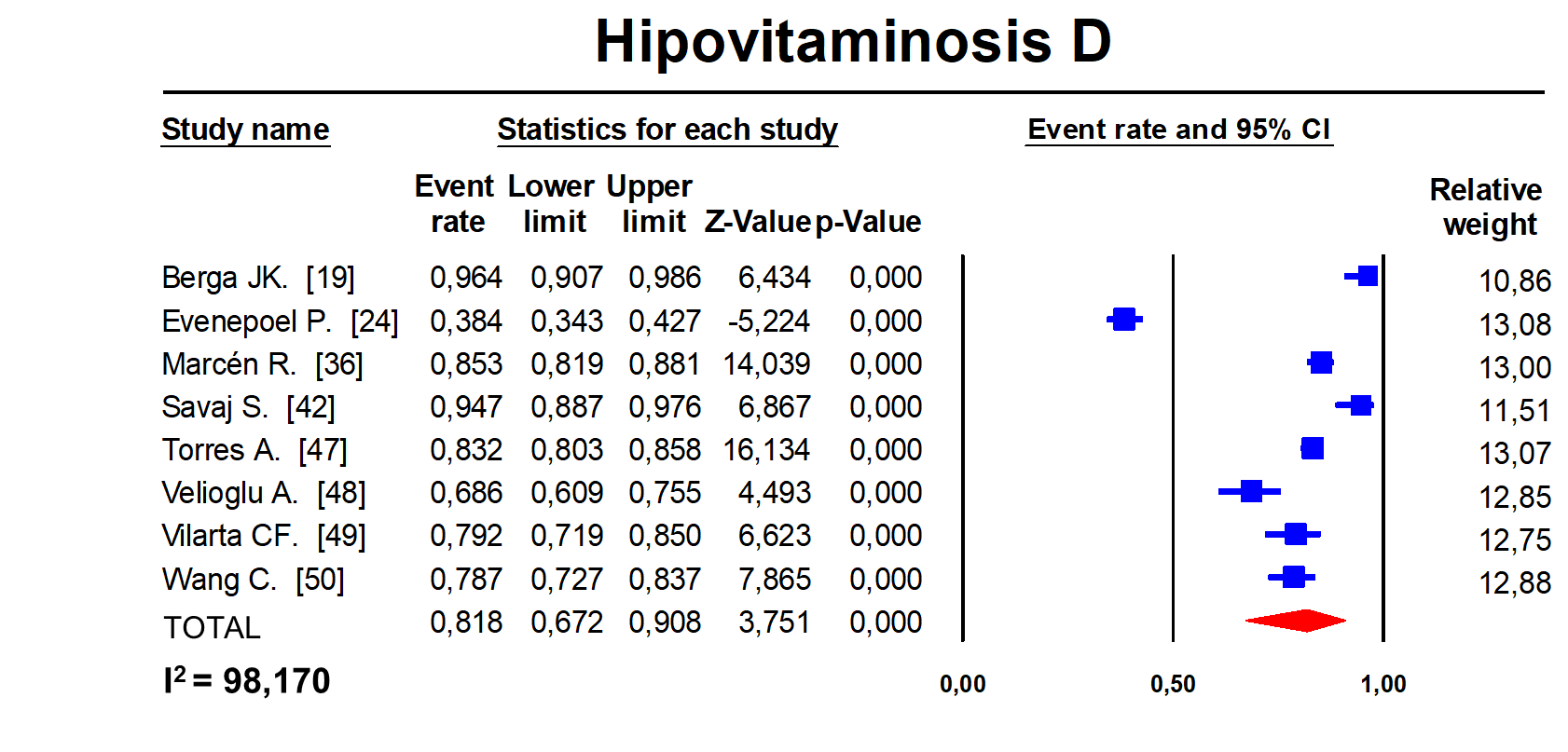


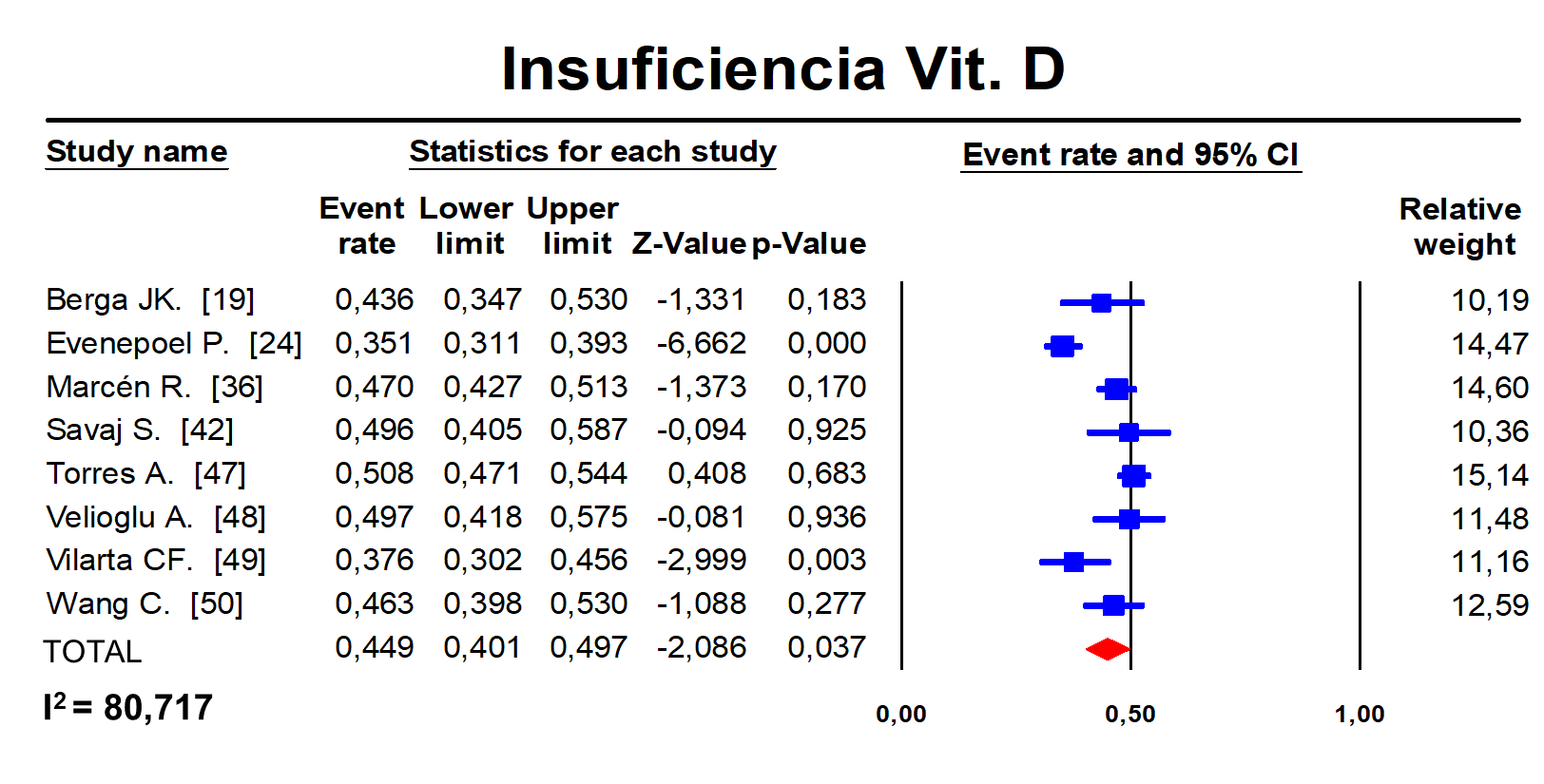


# **Figure S15.** Pooled prevalence estimates for vitamin D insufficiency.


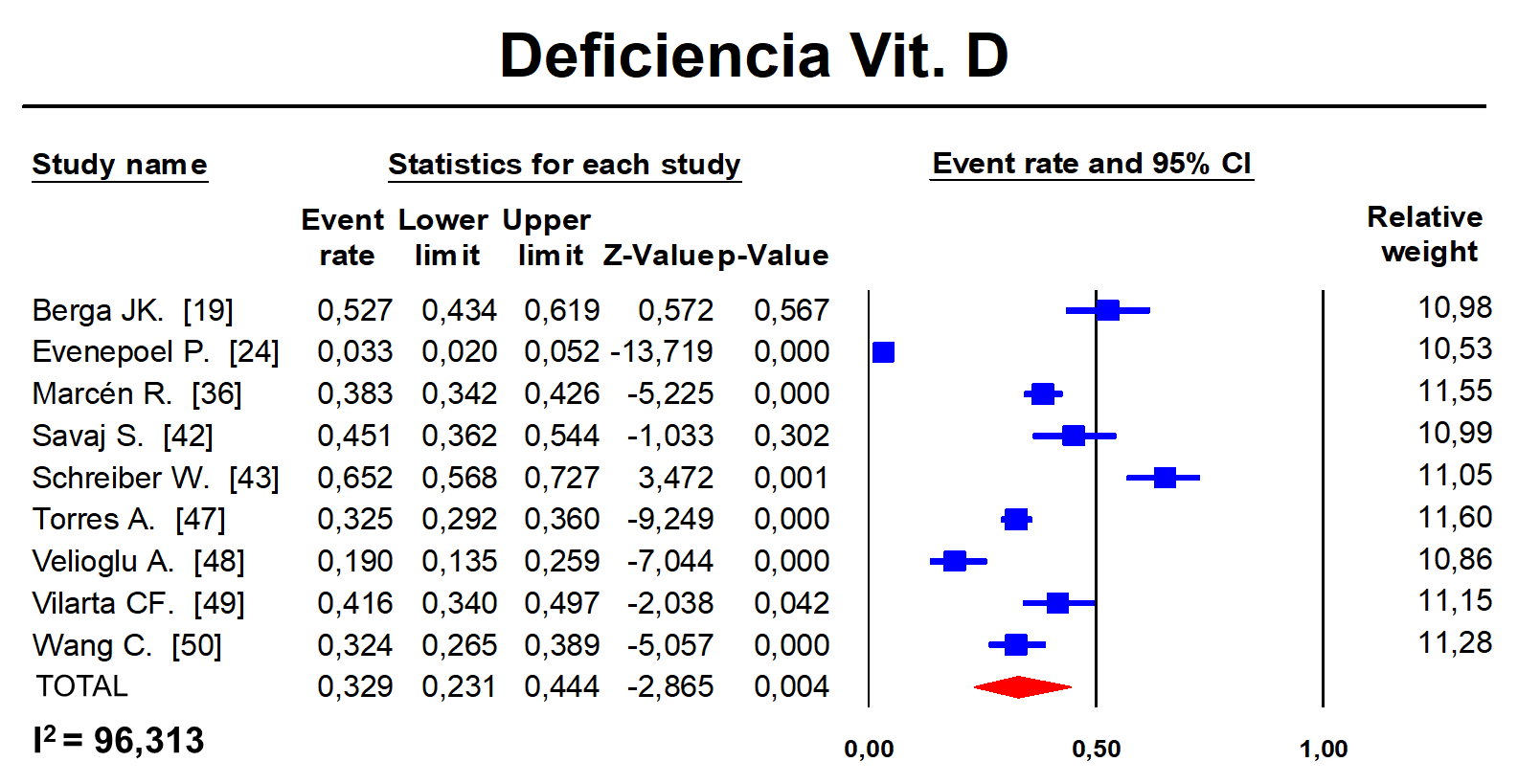


# **Figure S16.** Pooled prevalence estimates for vitamin D deficiency.

# **Figure S17**. Comparison of the prevalence of hypercalcemia after kidney transplantation versus the overall prevalence.

# **Figure S18**. Comparison of the prevalence of hyperparathyroidism after kidney transplantation versus the overall prevalence.

# **Figure S19**. Comparison of the prevalence of hyperuricemia after kidney transplantation versus the overall prevalence.

# **Figure S20**. Comparison of the prevalence of hipovitaminosis D after kidney transplantation versus the overall prevalence.

# **Figure S21**. Comparison of the prevalence of sarcopenia after kidney transplantation versus the overall prevalence.

# **Figure S22**. Comparison of the prevalence of low muscle mass after kidney transplantation versus the overall prevalence.

# **Figure S23**. Comparison of the prevalence of osteopenia after kidney transplantation versus the overall prevalence.

# **Figure S24**. Comparison of the prevalence of osteoporosis after kidney transplantation versus the overall prevalence.

# **Figure S25**. Comparison of the prevalence of fractures after kidney transplantation versus the overall prevalence.

# **Figure S26**. Comparison of the prevalence of gout after kidney transplantation versus the overall prevalence.
